# Quantifying the phase separation property of chromatin-associated proteins under physiological conditions using an anti-1,6-hexanediol index

**DOI:** 10.1186/s13059-021-02456-2

**Published:** 2021-08-17

**Authors:** Minglei Shi, Kaiqiang You, Taoyu Chen, Chao Hou, Zhengyu Liang, Mingwei Liu, Jifeng Wang, Taotao Wei, Jun Qin, Yang Chen, Michael Q. Zhang, Tingting Li

**Affiliations:** 1 MOE Key Laboratory of Bioinformatics, Bioinformatics Division and Center for Synthetic & Systems Biology, BNRist, School of Medicine, Tsinghua University, Beijing, 100084 China; 2 Department of Biomedical Informatics, School of Basic Medical Sciences, Peking University Health Science Center, Beijing, 100191 China; 3 Department of Cellular and Molecular Medicine, Institute of Genomic Medicine, University of California, La Jolla, San Diego, CA 92093 USA; 4 State Key Laboratory of Proteomics, Beijing Proteome Research Center, National Center for Protein Sciences (Beijing), Beijing Institute of Lifeomics, Beijing, 102206 China; 5 Laboratory of Proteomics, Institute of Biophysics, Chinese Academy of Sciences, Beijing, 100101 China; 6 National Laboratory of Biomacromolecules, Institute of Biophysics, Chinese Academy of Sciences, Beijing, 100101 China; 7 The State Key Laboratory of Medical Molecular Biology, Department of Molecular Biology and Biochemistry, Institute of Basic Medical Sciences, Chinese Academy of Medical Sciences, School of Basic Medicine, Peking Union Medical College, Beijing, 100005 China; 8 Department of Biological Sciences, Center for Systems Biology, The University of Texas, Richardson, TX 75080-3021 USA

## Abstract

**Background:**

Liquid-liquid phase separation (LLPS) is an important organizing principle for biomolecular condensation and chromosome compartmentalization. However, while many proteins have been reported to undergo LLPS, quantitative and global analysis of chromatin LLPS property remains absent.

**Results:**

Here, by combining chromatin-associated protein pull-down, quantitative proteomics and 1,6-hexanediol (1,6-HD) treatment, we develop Hi-MS and define an anti-1,6-HD index of chromatin-associated proteins (AICAP) to quantify 1,6-HD sensitivity of chromatin-associated proteins under physiological conditions. Compared with known physicochemical properties involved in phase separation, we find that proteins with lower AICAP are associated with higher content of disordered regions, higher hydrophobic residue preference, higher mobility and higher predicted LLPS potential. We also construct BL-Hi-C libraries following 1,6-HD treatment to study the sensitivity of chromatin conformation to 1,6-HD treatment. We find that the active chromatin and high-order structures, as well as the proteins enriched in corresponding regions, are more sensitive to 1,6-HD treatment.

**Conclusions:**

Our work provides a global quantitative measurement of LLPS properties of chromatin-associated proteins and higher-order chromatin structure. Hi-MS and AICAP data provide an experimental tool and quantitative resources valuable for future studies of biomolecular condensates.

**Supplementary Information:**

The online version contains supplementary material available at 10.1186/s13059-021-02456-2.

## Background

The cell nucleus is full of DNA, RNA, and proteins, which play important roles in regulating gene expression, DNA replication, cell division, etc. In order to perform multiple functions coordinately, functionally related biological macromolecules in the nucleus aggregate autonomously in a membrane-independent manner [1]. These nuclear condensates have been called “foci”, “hubs”, “clusters”, “speckles”, “factories”, or “phases”, but their assembly and regulation mechanisms remain unclear [2]. Recently, liquid-liquid phase separation (LLPS) has been proposed as one of possible mechanisms underlying the formation of nuclear condensates [3].

Many chromatin-associated proteins have been shown to undergo phase separation in vitro or/and in vivo, including RNA polymerase II [4, 5], mediator complex subunits [6], heterochromatin proteins 1 (HP1) [7, 8], and Polycomb protein chromobox 2 (CBX2) [9, 10]. Generally, LLPS proteins are identified by non-quantitative or low-throughput methods such as droplet roundness/fusion, immunofluorescence (IF), and fluorescence redistribution after photobleaching (FRAP) [11]. As a consequence, it is difficult to compare the LLPS properties of different proteins, although molecular dynamics and biological functions of these euchromatin- and heterochromatin-associated proteins differ considerably. In addition, the biomolecular condensation is closely related with molecular concentration, the presence of chaperone molecules and inter-/intra-molecular interaction with proteins and nucleic acids. Therefore, it is necessary to develop high-throughput method to better understand the composition and kinetics of nuclear condensates at physiological concentration in a systematic way.

The occurrence of LLPS and formation of condensates has been attributed to weak, dynamic multivalent interaction among molecules, including pi-pi interaction, charge-charge interaction, cation-pi interaction, and dipole-dipole interaction [1]. The aliphatic alcohol 1,6-hexanediol (1,6-HD) is widely used for disrupting LLPS condensates in vitro and in cell. It contains a hydrophobic group that is composed of 6 hydrogenated carbon atoms, which interfere with the hydrophobic interactions, and consequently affect hydrophobicity-dependent LLPS condensates [12, 13]. Furthermore, compared with another commonly used detergent, SDS, which consists of twelve hydrogenated carbon atoms, 1,6-HD is sufficiently weak to only disrupt LLPS condensates, without affecting either solid-like assemblies [14] or membrane-bounded organelles [15]. Moreover, a recent study reported that following 1,6-HD treatment of MCF-7 cells, the ChIP-seq peak signal for transcription factor (TF) GATA3 was significantly weakened, while the peak signal for another TF ER remained unchanged [16], suggesting that chromatin binding proteins exhibit protein-specific sensitivities to 1,6-HD treatment, thus affecting their ability to bind to DNA. This protein-specific sensitivity provides a valuable opportunity to quantitatively measure LLPS properties of nuclear condensates in their endogenous state and physiological abundance.

Capturing chromatin-associated proteins, especially those involved in nuclear condensates through LLPS, is challenging. Chromatin-associated proteins such as transcription factors or mediators are expressed at lower levels compared with constitutive proteins such as histones. Furthermore, nuclear condensates concentrate their components proximal to chromatin via weak and dynamic multivalent interactions [17]. However, current methods available for genome-wide capturing chromatin-associated proteins are too hash to maintain dynamic interactions. For instance, for CHEP (chromatin enrichment for proteomics), cells are usually washed using 4% SDS and 8 M urea [18], while for DEMAC (density-based enrichment for mass spectrometry analysis of chromatin), high speed centrifugation for at least 48 h is needed [19]. Thus, new methods capable of effectively capturing chromatin-associated proteins related to condensates are urgently required.

Here, we developed a method called Hi-MS that combines Hi-C based chromatin-associated protein pull-down, quantitative proteomics, and 1,6-HD treatment. Using Hi-MS, we defined quantitative and global measurement that reflects LLPS properties of chromatin-associated proteins. As LLPS has also been proposed as one of possible mechanisms to mediate chromosome compartmentalization [20, 21]. We analyzed chromatin organization changes after 1,6-HD treatment using BL-Hi-C. By applying these methods, we obtained a first global view of LLPS properties of nuclear condensates and chromatin organization in their endogenous state.

## Results

### Development and validation of Hi-MS

**Fig. 1:**
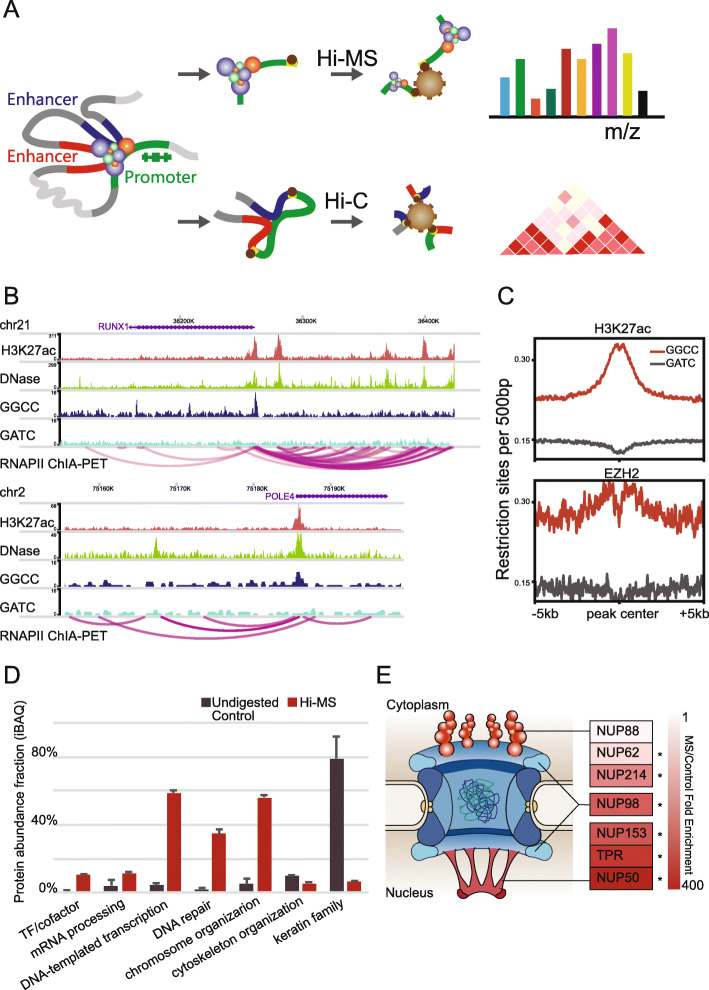
Hi-MS effectively enrich chromatin-associated proteins. **A** Schematic of Hi-MS. **B** The examples of GGCC distribution in the gene promoter region. Top, RUNX1; bottom, POLE4. These two regions were selected as represents of super enhancer and typical enhancer [23]. **C** The distribution of human genome sequence GATC and GGCC proximate to H3K27ac and EZH2 binding peaks. **D** Chromatin-associated proteins are effectively enriched by Hi-MS compared with undigested control. **E** MS/control fold enrichment of NUPs agreed with their spatial location. NPC model was adapted from Ref [24]

In order to quantify chromatin-associated protein changes after 1,6-HD treatment, we developed a method that effectively captures chromatin-associated proteins in situ (Fig. 1A). To enrich regulatory proteins, we targeted gene promoter regions based on genome sequence preference. As shown in Fig. 1B, GGCC is a nucleotide sequence enriched in gene promoter regions. We previously developed a method called BL-Hi-C [22], which uses restriction endonuclease *Hae*III to cut at GGCC sites to enrich cis-regulatory elements of gene promoters, including both activated elements marked by H3K27ac and repressed elements marked by EZH2 (Fig. 1C). This procedure is relatively gentle, and most of the chromatin-associated proteins are well preserved. Here, we used a protocol based on BL-Hi-C to enrich chromatin-associated proteins. Briefly, we crosslinked cells using 1% formaldehyde and then digested the genome by *Hae*III; then, the digested DNA fragment ends were ligated via biotinylated bridge linker. Next, we sonicated the cells, and the biotinylated linker/DNA/protein complexes were captured by magnetic streptavidin-beads, before label-free quantitative mass spectrometry (MS) analysis. We named this method Hi-MS corresponding to the name of Hi-C (Fig. 1A). We used Hi-MS to extract chromatin-associated proteins in K562 cell line, which is a widely used cell line with an extensive public omics data set. Typically, 10^8^ cells are required for genome-wide chromatin protein capture methods [18, 19], while for Hi-MS analysis, 10^7^ cells are sufficient. Importantly, protein abundance fraction of TFs and cofactors were enriched 5-fold in the Hi-MS sample compared to undigested control samples (Fig. 1D). Other nuclear proteins involved in mRNA processing, transcription, DNA repair, and chromosome organization were enriched 4-15 folds, while proteins involved in cytoskeleton organization represented by KRTs were significantly reduced (Fig. 1D).

Nuclear pore complex (NPC) is a well-studied multi-protein complex with components located at both sides of nuclear membrane [24]. Components located on the nucleus side have more opportunities to bind to DNA. Hence, we determined the efficiency of our Hi-MS method in enriching chromatin-associated proteins by analyzing the components of NPC. As shown in Fig. 1E, cytoplasmic filament components, which locate on the cytoplasm side of NPC, including NUP214, NUP88, and NUP62, showed an average abundance enrichment of 102. In comparison, nuclear basket components, which locate on the nuclear side of NPC, including TPR, NUP50, and NUP153, showed an average abundance enrichment of 339. The nuclear/cytoplasmic ring component NUP98, which locates in the middle of the NPC, showed a fold enrichment of 262. Together, these results indicated that Hi-MS is a sensitive and efficient method to capture chromosome-associated proteins in situ.

### Proteins exhibit different sensitivities to 1,6-HD treatment

In order to effectively measure the sensitivity of chromatin-associated proteins to 1,6-HD treatment by quantitative proteomics, we first titrated the concentration of 1,6-HD by testing local distribution of several proteins using immunofluorescence. It was previously reported that in HeLa cells, stress granules (SG) and P bodies (PB) can be dissolved following 1,6-HD treatment [25]. We based our incubation time on this method and tested the dissolution of MED1/FUS puncta at different concentrations of 1,6-HD in K562 cells. As shown in Additional file 1: Figures S1A-B, 10% 1,6-HD effectively dissolved most MED1 puncta. However, no obvious FUS puncta can be seen in the cell, with increasing 1,6-HD concentrations, the FUS protein gradually translocated into the cytoplasm. As 1,6-HD has the capability to dissolve the channel in NPC [26], we speculate that this translocation occurred because the NPC channel was destroyed.

It was also reported that after the dissolution of stress granules, smaller stress granule-like structures re-accumulate in a significant percentage of cells. However, hypotonic medium effectively alleviates this re-accumulation [14], probably because cells swell slightly in hypotonic conditions, thus reducing the local protein concentration. Based on this report, we mixed 30% 1,6-HD aqueous solution with normal K562 cell culture medium, and obtained a 2/3 dilution of medium with 10% 1,6-HD. To evaluate the sensitivity of proteins to this hypotonic 1,6-HD treatment, we used the cytoplasmic/nuclear proteins ratio as a measurement of sensitivity to 1,6-HD treatment. As shown in Additional file 1: Figures S1C-D, the order of sensitivity was MED1 > FUS > EZH2 > H3.

Together, these results indicated that each protein is characterized by a protein-specific sensitivity to 1,6-HD treatment, which allows subsequent quantitative measurement of this sensitivity using Hi-MS.

### Evaluating 1,6-hexanediol sensitivity of proteins using AICAP

**Fig. 2:**
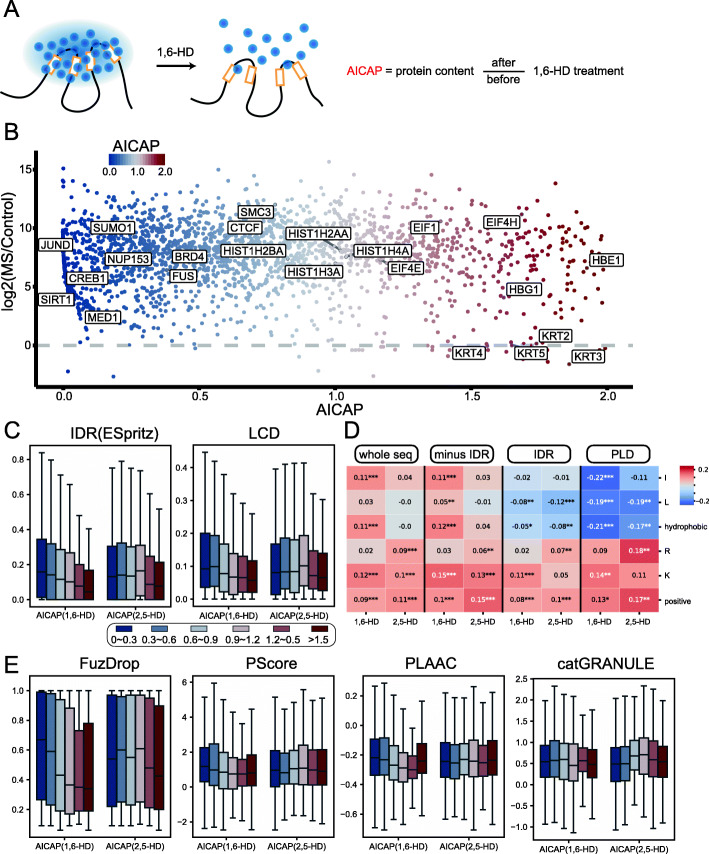
AICAP consists with known physicochemical properties involved in phase separation. **A** Schematic of 1,6-HD treatment and definition of AICAP. **B** Scatter plot of proteins with AICAP values 0~2 captured by Hi-MS. **C** The disorder compositions of 6 groups of proteins. IDR, intrinsically disorder region; LCD, low complexity domain. **D** The Spearman correlation between AICAP and corresponding residue percentage in proteins with AICAP < 1. Each column panel refers to residue percentage in different regions (whole sequence, whole sequence minus IDR, IDR, and PLD). PLD, prion-like domain. **E** The LLPS predictor scores of 6 groups of proteins

In this section, we set out to quantitatively measure the sensitivity of chromatin-associated proteins to 1,6-HD treatment by Hi-MS. As demonstrated earlier, proteins exhibit different sensitivities to 1,6-HD treatment. With the quantified protein amount before and after 1,6-HD treatment, we defined an anti-1,6-HD index of chromatin-associated proteins (AICAP) (Fig. 2A). This index quantitatively reflects the sensitivity of every chromatin-associated protein to 1,6-HD treatment. Additional file 2: Table S1 provides a complete list of AICAP value for all captured proteins.

We prepared three batches of biological duplication samples using Hi-MS, each batch containing three treatments, 1,6-HD-, 1,6-HD+, and undigested control. As shown in Figure S2A, the same treatment in different biological duplications can be well clustered, indicating high reproducibility of 1,6-HD treatment. We obtained the AICAP values for 3228 chromatin-associated proteins through mass spectrometry (MS) analysis (Additional file 2). Lower AICAP values indicate that the corresponding proteins are more sensitive to 1,6-HD treatment. As shown in Fig. 2B, proteins that can undergo LLPS such as FUS, SUMO1, MED1, and YY1 showed low AICAP values; the chromatin architecture protein CTCF and SMC3 showed relatively high AICAP values but still lower than 1.0; proteins of the histone family showed AICAP values around 1.0, while KRTs and EIFs and other typical cytoplasmic proteins showed AICAP values above 1.0. Because most of AICAP>1 protein comes from the cytoplasm (Additional file 1: Figure S2B), we pay more attention to proteins with AICAP value 0–1 in the following studies.

To test the robustness of our AICAP, we treated different batches of cultured cells, performed different types of digestion (in gel or in solution) and analyzed on different types of mass spectrometers (Q Exactive or Orbitrap Fusion Lumos Plus). As shown in Additional file 1: Figure S2C-D, the AICAPs generated by the two batches of experiments significantly correlated with each other. The Spearman correlation coefficient of two batches reached 0.533 with the *p*-value 1.5e− 145. More proteins were obtained using in-solution digestion (condition 2 in method details), so the MS data used in following analysis were from condition 2. We further compared the AICAP of common nuclear condensates proteins and found that the AICAP of proteins in the same condensate correlated well. The Spearman correlation coefficient of nuclear condensates proteins between two batches reached 0.61, with a *p*-value of 2.2e− 16 (Additional file 1: Figure S2D). Together, these results demonstrated that the AICAP values generated by our Hi-MS method were robust.

### AICAP consists with known physicochemical properties involved in phase separation

It was reported that 1,6-HD as well as 2,5-HD, an isomer of 1,6-HD, can impose global effect on cells such as nucleic compaction [27]. According to previous cases, 2,5-HD is not as strong as 1,6-HD in dissolving the hydrophobicity-dependent condensate and therefore considered as negative control for 1,6-HD [13]. To confirm that observed AICAP is consequence of specific disruption against hydrophobic interactions rather than non-specific global effect, we conducted a parallel Hi-MS experiment using 2,5-HD treatment. 2,5-HD treated samples can also be clustered from untreated control, further indicating the robustness of Hi-MS (Additional file 1: Figure S2E). There was little correlation between AICAP of 1,6-HD and 2,5-HD when common condensate related proteins were considered (Additional file 1: Figure S2F).

Previous researches have studied the protein structural features related with LLPS potential. Briefly, multivalent interactions can be mediated by intrinsically disordered regions (IDRs) or low complexity domains (LCDs). Residue composition has also been reported to play important roles in multivalent interactions. In this study, we found that lower AICAP proteins possess more IDRs and LCDs (Fig. 2C), consistent with previous reports. After further analyzing the correlation between AICAP and amino acid composition in ordered/disordered region, we found that AICAP is negatively correlated with hydrophobic residue content (such as I and L) in IDR and PLD and positively correlated with positive charged residues (such as K and R) (Fig. 2D, Additional file 1: Figure S3). Because the lower AICAP proteins were more sensitive to 1,6-HD treatment, our observation suggested that proteins with more hydrophobic residues in disordered regions were more sensitive to 1,6-HD. However, this relationship was not obvious or even opposite when we focus on the residue composition of ordered region or whole protein (Fig. 2D, Additional file 1: Figure S3), consistent with previous study emphasizing the importance of fraction and distribution of hydrophobic residues in disordered proteins [28].

LLPS can be promoted by various kinds of interactions, many computational approaches have been developed to predict protein’s probability to undergo LLPS [29]. FuzDrop [30] evaluates protein’s droplet-forming propensity based on biophysical principles driving phase separation, especially non-specific side-chain interactions. PScore was developed based on pi-pi interaction frequency to screen LLPS proteins [31]. PLAAC was widely used to predict probability of prion-like domains (PLDs), which were often found in known LLPS proteins [32]. catGRANULE was initially trained to predict inappropriate liquid phase separation based on yeast proteome [33]. Hence, to better evaluate the relationship between AICAP and protein’s propensity to be phase separated, we compared AICAP with these phase separation prediction tools (Fig. 2E). We found that AICAP negatively correlates with FuzDrop score. Similar but not as strong negative correlation was observed for PScore and PLAAC prediction, which was reasonable because pi-pi interactions and PLDs account for an even smaller subset of condensate-promoting interactions, while catGRANULE did not exhibit clear correlation with AICAP. Importantly, the AICAP of 2,5-HD experiment did not reproduce similar correlation (Fig. 2C, E), confirming that AICAP is a consequence of specific disruption effect by 1,6-HD rather than non-specific global effect.

### Extensive comparison between AICAP and proteins in biomolecular condensates

**Fig. 3:**
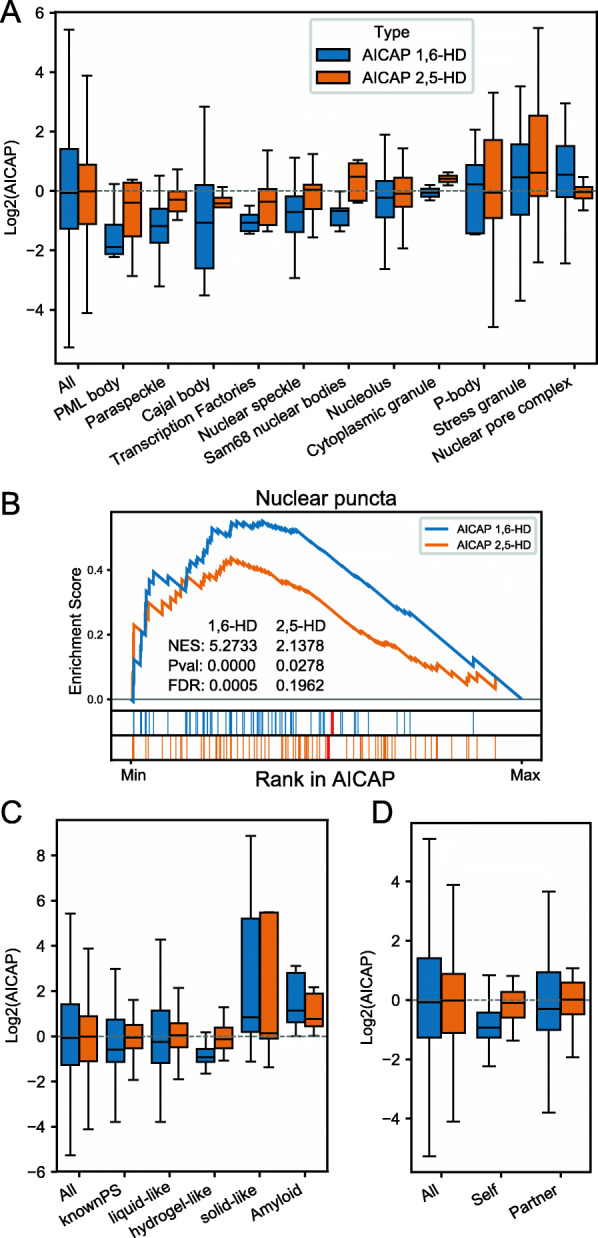
Comparison between AICAP and proteins in biomolecular condensates. **A** Distribution of AICAP values of proteins in different condensates. **B** GSEA enrichment of “Nuclear puncta” [35] using AICAP ranked proteins. **C** Distribution of AICAP values of proteins annotated to be of different material states. **D** Distribution of AICAP values of self-assembling (Self) and partner-dependent (Partner) proteins

Next, we examined the relation between AICAP and protein’s involvement in biomolecular condensates (Additional file 3). Generally, the biomolecular condensates can be classified into two classes. The first class is typical nuclear condensates, including nuclear speckles, paraspeckles and PML bodies, most proteins in this class exhibit low AICAP values with 1,6-HD treatment (Fig. 3A). While the other class is condensates closely associated with cytoplasm, including stress granule (typical cytoplasm condensates), nuclear pore complex (condensates between cytoplasm/nuclear), or ribosomes/nucleolus whose main components (RPLs, RPSs) shuttle between the cytoplasm and nucleoli [34]. Many proteins in this class exhibit high AICAP values (Fig. 3A), which may enter the nucleus after NPC channel disruption [26]. Since most of these proteins have little relationship with chromatin, so we paid more attention to the first class condensates in this study. As shown in Fig. 3A, compared to 1,6-HD, the changes caused by 2,5-HD to nuclear condensates was much weaker. Fewer proteins exhibit decreased or increased association with chromatin, while more proteins remain unchanged, suggesting 2,5-HD cause weaker disruption effect against interactions within nuclear condensates than 1,6-HD.

OpenCell is a newly developed resource that provides endogenous protein subcellular location information based on confocal images of CRISPR-edited cell lines [35]. Using Gene Set Enrichment Analysis (GSEA) analysis, we observed that nuclear puncta proteins annotated by OpenCell were significantly enriched at the low AICAP end (Fig. 3B, Additional file 3). In contrast, AICAP of 2,5-HD showed less significance, which is consistent with changes in various condensates aforementioned.

Biomolecular condensates can exhibit distinct material states, which are important in cellular functions and pathogenesis. It was previously reported that 1,6-HD cannot disrupt solid-like condensate [14]. We extract material state annotations from PhaSepDB [36] and AmyPro database [37], the analysis showed that proteins reported to be liquid-like or hydrogel-like possess lower AICAP than average, while solid-like or amyloid fiber-forming proteins possess high AICAP on the contrary (Fig. 3C). This difference coincides with previous observations.

Driver/client theory is widely used to explain biomolecular condensate formation, in which drivers can spontaneously undergo phase separation while clients can be recruited into corresponding condensates. However, there is still a lack of experimental based drivers and clients list. Here, we classify known phase separation proteins into self-assembling and partner-dependent ones based on experimental evidences extracted from PhaSepDB [36] (Additional file 3, “Method details”). We examine AICAP of self-assembling and partner-dependent proteins and found that both display lower AICAP values than average, while self-assembling proteins possess even lower AICAP (Fig. 3D). The difference suggests that self-assembling proteins depend more on hydrophobic interactions.

Together, 1,6-HD cause stronger disruption effect against interactions within biomolecular condensates than 2,5-HD, especially liquid condensate and the self-assembling proteins.

### AICAP values were verified by independent ChIP-seq data

**Fig. 4:**
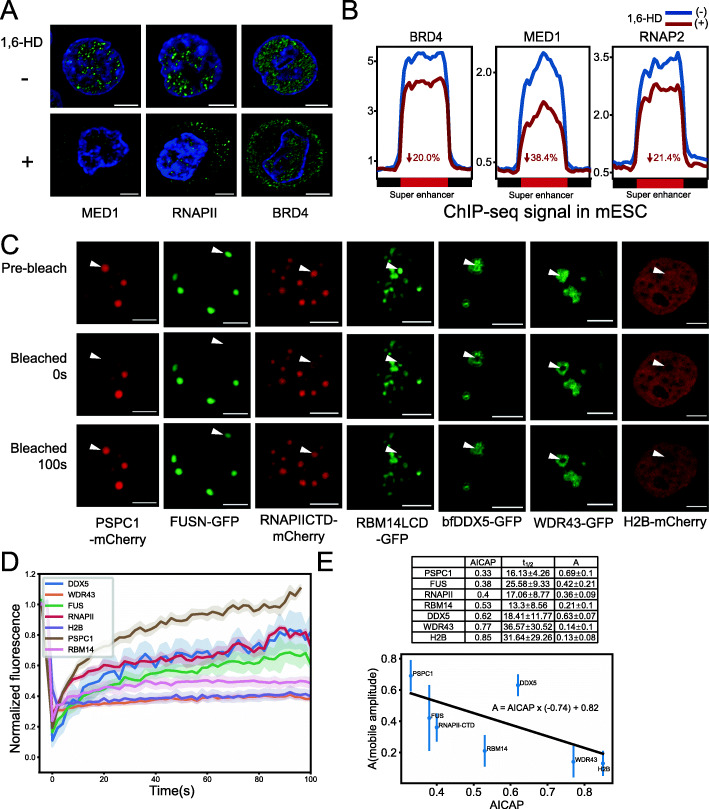
Experimental validation of AICAP. **A** Immunofluorescence of MED1, RNAPII, and BRD4 before (−) and after (+) 1,6-HD treatment. Scale bar indicates 5 μm. **B** ChIP-seq signal enrichment of BRD4, MED1, and RNAPII at regions defined as super enhancer in mESC before (1,6-HD−) and after (1,6-HD+) 1,6-HD treatment. The decreased percentage of signal is noted. Data resource [6]. **C** FRAP experiments on 7 proteins in HeLa cells. Scale bar indicates 5 μm. **D** FRAP curves of proteins in C. **E** Calculated parameters of FRAP experiments using chemical interaction model (top). The mobile fraction was fitted using linear regression (bottom)

To further test the relationship between AICAP and nuclear condensate, we compared the AICAP values of proteins with independent ChIP-seq data. BRD4, MED1, and RNAPII were reported to form LLPS condensate in mES cells [6]. Immunofluorescence experiments confirmed that in K562 cells, these three proteins exhibit puncta of varying number and sizes. The use of 1,6-HD treatment can weaken these puncta, and the proteins are further translocated to the cytoplasm (Fig. 4A). In previous published data [6], the ChIP-seq data of BRD4, MED1 and RNAPII before and after 1,6-HD treatment were compared in mESC cells. The result showed that at the super enhance (SE) region of Klf4, the occupancy levels of BRD4, MED1 and RNAPII were reduced by 44%, 80%, and 56%, respectively. We reprocessed their ChIP-seq data, and calculated the reads density at all SEs before and after 1,6-HD treatment, and found that the occupancy levels of BRD4, MED1 and RNAPII were reduced by 20%, 38.4%, and 21.4%, respectively, which is similar to that of Klf4 (Fig. 4B). The results obtained from that ChIP-seq data were consistent with the AICAP values, which were 0.537 for BRD4, 0.072 for MED1, and 0.401 for RNAPII. Moreover, in droplet disturbing assays, the droplet size of MED1-IDR decreased more than that of BRD4-IDR with increasing NaCl concentration [6]. These differences between MED1 and BRD4 agreed with their AICAP values, which further revealed relationship between AICAP and LLPS property.

### Proteins with high mobility displayed low AICAP values

FRAP is a regular assay for studying protein dynamics. In order to find out the relationship between AICAP and protein mobility, we performed a batch of protein FRAP experiments (Fig. 4C). FRAP curves are fitted with a chemical interaction model in which amplitude of mobile population (A) and half-time to recover (*t*_1/2_) were calculated (Fig. 4D, E, “Method details”). Amplitude of mobile population (A) reflects the fraction of mobile components recovered over time. As shown in Fig. 4C and D, after bleaching, proteins such as PSPC1, FUSN, and RNAPII-CTD can recover to about 40~70% of original intensity, while other proteins like RBM14LCD, WDR43, and H2B can only recover to about 10~20% of original intensity, displaying lower mobility. We fitted amplitude of mobile population (A) as a function of AICAP using linear regression, the result indicates that A negatively correlated with AICAP (Fig. 4E). These results demonstrated that the proteins with lower AICAP are related with higher mobility under intracellular condition.

### Active transcriptional chromatin regions and associated proteins are more sensitive to 1,6-HD treatment

Recent studies have suggested an important role of LLPS in chromatin organization, such as chromatin compaction and heterochromatin formation [7, 8]. Following 1,6-HD treatment, chromatin-associated proteins dissociate from the chromatin at various amounts. To study the effect of this dissociation by 1,6-HD on the higher-order chromatin organization, we constructed BL-Hi-C libraries in untreated K562 cells or flowing 1,6-HD treatment. For each treatment, we constructed three biological replicates. As shown in Additional file 1: Figure S4A, the clustering data showed that the effect of 1,6-HD on chromatin organization is highly reproducible, and BL-Hi-C is capable of detecting the cis-unique DNA interactions with higher efficiency (Additional file 1: Figure S4B-D, additional file 4).

**Fig. 5:**
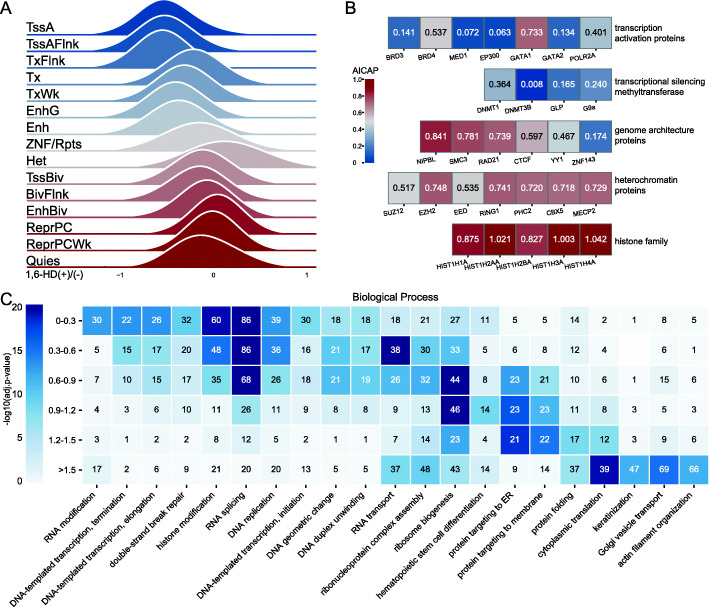
Active transcriptional chromatin regions and associated proteins are more sensitive to 1,6-HD treatment. **A** Intra-chromosome interactions of 15 types epigenome chromatin states after 1,6-HD treatment. **B** The AICAP values of hallmark proteins in different functional categories. **C** Gene ontology biological process enrichment analysis of proteins. Proteins were divided into 6 groups based on AICAP (*Y*-axis). Number of proteins was noted in the corresponding cell

Using BL-Hi-C data, we tested the DNA-DNA interaction changes of functional DNA elements to explore their sensitivity to 1,6-HD treatment. Functional DNA elements were characterized by 15 types of epigenome chromatin states [38]. As shown in Fig. 5A, the intra-chromosome interaction of active transcription regions, including active TSSs and enhancers were most affected, while repressed chromatin regions/areas, including bivalent regions and heterochromatin were less affected. Moreover, the AICAP of hallmark proteins involved in these regions showed good consistency with the sensitivity to 1,6-HD treatment (Fig. 5B). Transcription activation and regulatory proteins exhibit low AICAP values, while heterochromatin proteins and chromatin structural proteins exhibit high AICAP values.

Apart from these hallmark proteins, we compared the functions of all proteins with different AICAP values (Additional file 5). We divided proteins into six groups based on AICAP, and proteins in each group were clustered according to categories of “biological process” (BP, Fig. 5C), “molecular function” (MF, Additional file 1: Figure S4E), or “cellular component” (CC, Additional file 1: Figure S4F). As shown in Fig. 5C, the main enrichment BP terms within group “0-0.3” constituted RNA modification/splicing, DNA transcription, histone modification, and the corresponding CC terms of RNA pol II/TF complex, nuclear speckle, all of which are active regulatory processes. The enriched BP terms near the AICAP value of 1.0 are primarily constitutive nuclear condensate represented by ribosomes. Proteins with AICAP values above 1.0 are related to protein translation, folding, and transport, most of which are usually located in the cytoplasm.

Together, the results demonstrate that the active/regulatory chromatin components, both protein (trans) and DNA (cis), exhibit more hydrophobicity-dependent LLPS properties than the repressed/structural chromatin components.

### A/B compartment of chromatin exhibited different sensitivities to 1,6-HD treatment

**Fig. 6:**
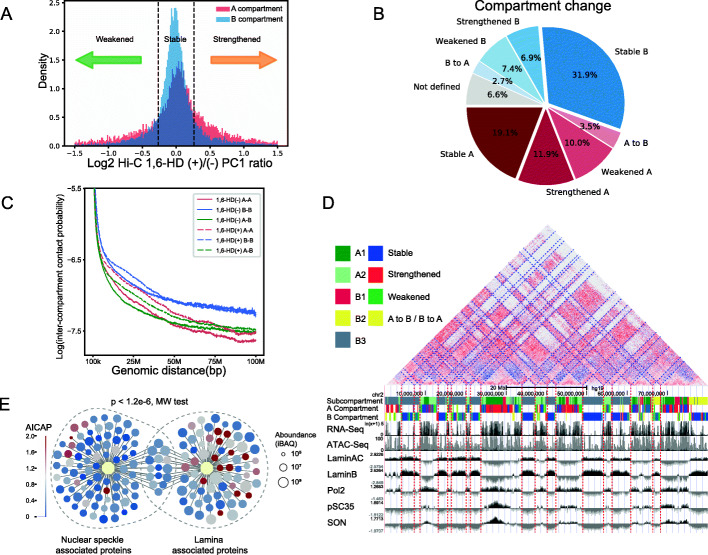
A/B compartment of chromatin exhibited different sensitivities to 1,6-HD treatment. **A** A (red) and B (blue) compartments can be classified into 4 compartment change-types as strengthened, stable, weakened based on their PC1 value ratio (1,6-HD+/−). 20% was chosen as the threshold for distinguishing stability or not. **B** The fraction of four kinds of compartment change-types. **C** Contact probability between compartments along genomic distance. **D** Examples of strengthened/stable compartments and corresponding nuclear speckle/lamina TSA-seq [39] plots. The plotting of log2 ratio of TSA read density versus input read density was used to measure the distance from a chromatin region to a specific nuclear condensate. Chr2, 0–80 M. **E** AICAP values of nuclear speckle and lamina associated proteins. Data resource [40, 41]

By using Hi-C technique to analyze chromatin organization, chromatin is partitioned into two compartments termed “A” and “B”, which correlate with transcriptional activation (A compartment) or repression (B compartment). Compartments are further partitioned into topologically associating domains (TADs) and chromatin loops. Here, in this section, we studied the sensitivities to 1,6-HD treatment in the scope of 3D chromatin structure. Following 1,6-HD treatment, compartment changes were subdivided into four compartment change-types based on the changes of the compartment strength (PC1 value, “Method details”): strengthened, stable, weakened, and flipped compartment (Fig. 6A, B). As shown in Fig. 6A and B and Additional file 1: Figure S5A, the B compartment is more stable after 1,6-HD treatment compared to the A compartment. The changes of the inter-compartment interaction also showed that compartment A is more dynamic than B (Fig. 6C, D). We found that the number of interactions between A-A compartments, even at distances as long as 100 Mb, significantly increased. In contrast, although short distance B-B interactions also increased, long distance B-B interactions were stable (Fig. 6C, D).

We analyzed the AICAP value of proteins associated with nuclear speckle and nuclear lamina, which corresponding to A and B compartments, respectively [42]. Previous studies have used proximate labeling to capture proteins located proximally to the nuclear speckle and lamina and obtained high confidence subsets by comparing appropriate control samples [40, 41]. As shown in Fig. 6E, proteins included in nuclear speckles exhibit low AICAP values, characterized by a median value of 0.50. In comparison, proteins included in nuclear lamina possess high AICAP values, with a median value of 0.81.

Interestingly, we found that compartment changes were constrained by neighbor compartment. For the strengthened and stable compartments, their neighbors were often the same types of compartments, while for the weakened compartments, their neighbors were often different types of compartments (Additional file 1: Figure S5C). Meanwhile, following 1,6-HD treatment, the compartments undergoing a conversion from A to B were usually surrounded by B compartments, while the compartments undergoing B to A conversion were commonly surrounded by A compartments (Additional file 1: Figure S5B-C).

Together, these results indicate that compartment A are more sensitive to 1,6-HD treatment, which is in agree with the changes of chromatin at different states aforementioned. This result is also consistent with one recent study showing that heterochromatic regions exhibit stronger internal attractions than euchromatin [43].

### Active chromatin TADs/loops are more sensitive to 1,6-HD treatment

**Fig. 7:**
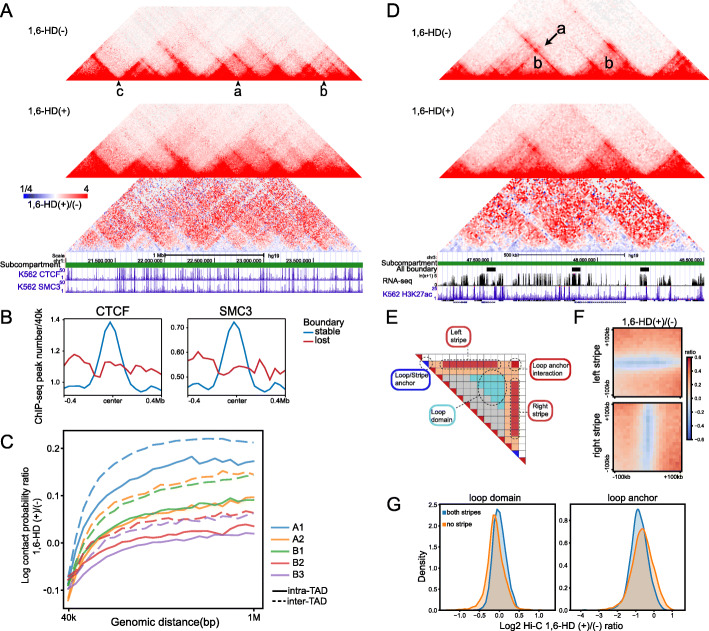
Active chromatin TADs/loops are more sensitive to 1,6 HD treatment. **A** Examples of stable/lost TAD boundaries. example “a, b” lost, example “c” stable. **B** Aggregation analysis of CTCF/SMC3 ChIP-seq peaks at stable/lost TAD boundaries. **C** Intra-/inter-TAD interaction changes (1,6-HD(+)/(−) ratio) in different subcompartments. Interactions between different subcompartments were skipped. **D** Examples of loop domain "b", and DNA stripe "a" after 1,6-HD treatment. **E** Schematic illustration of loop anchor interaction, loop domain, DNA stripe, and stripe anchor. **F** Left and right stripe signal aggregation at 10 kb resolution. **G** Aggregation analysis of loop domain (left) and loop anchor interaction (right) signal changes after 1,6-HD treatment. “both/no stripe” indicates both or no overlap between loop anchor and stripe anchor

To further evaluate the dynamics of finer chromatin structure, we next investigated the chromatin organization changes at TAD and loop level following 1,6-HD treatment. The inter-TAD interaction increased significantly at a number of sites (example “a, b” in Fig. 7A), which resulted in the loss of 15.6% of the TAD boundaries (Additional file 1: Figure S6A). Compared with the lost boundaries, CTCF and cohesin (SMC3) peaks were significantly enriched at the stable boundaries (Fig. 7B), and tandem strong CTCF peaks coexist in the same boundary (example “c” in Fig. 7A). Furthermore, the sensitivity of TAD to 1,6-HD treatment showed a subcompartment-dependent manner. The A/B compartment can be divided into five subcompartments namely A1, A2, B1, B2, and B3, representing different transcription activity [44]. A1 and A2 were both characterized by active chromatin with A1 most enriched in active histone modifications. B1, B2, and B3 were inactive chromatin and B1 is marked by bivalent histone modifications. As shown in Fig. 7C, the order of intra and inter-TAD interactions increment was A1 > A2B1 > B2B3. This observation coincides with previous report that A1, A2, and B1 are more dynamic than B2 and B3 [45]. Considering that different subcompartments were associated with different nuclear condensates [39], the sensitivity to 1,6-HD provides an evidence that chromatin conformation is closely related to composition of surrounding environment.

1,6-HD treatment caused drastic changes on DNA loops. The overall strength of the loops decreased 60% on average (Additional file 1: Figure S6B), and 61.5% of all DNA loops disappeared after 1,6-HD treatment (Additional file 1: Figure S6C), which was much more than that of TAD boundary. DNA stripe is a subunit of DNA loop which is considered to be the prototypes of cohesin extrusion, and nearly 80% of stripe domains were associated with active enhancers in mouse B cells [46]. After 1,6-HD treatment, both left and right DNA stripes were visibly weakened (example “a” in Fig. 7D, F). However, interactions surrounding the DNA stripes (loop domain, example “b” in Fig. 7D) appeared to be increased (Fig. 7G). Since stripe anchors often locate coincide with loop anchors, DNA loops can be divided into two types, which are loops carrying two or none DNA stripes. We therefore tested the stability of these two types of DNA loops. As shown in Fig. 7G, DNA loops containing DNA stripes (both stripes) weakened more than the loops not containing DNA stripes (no stripe).

In summary, these results strongly indicated that the transcriptionally active 3D chromatin organizations are more sensitive to 1,6-HD treatment, including compartments, TADs, and loops. These findings were consistent with the protein AICAP in corresponding chromosomal regions.

## Discussion

Developing a systematic experimental methodology to identify and characterize biomolecular condensates is crucial for further development of the LLPS field. In this study, we disrupted hydrophobicity-dependent LLPS properties by 1,6-HD treatment, then developed Hi-MS and performed Hi-C to quantify changes in both chromatin binding proteins and higher-order chromatin structure in K562 cell. Next, we systematically compared AICAP with known physicochemical properties involved in phase separation.

Through analyze protein sequence and residue composition, we found that proteins with low 1,6-HD AICAP values exhibit higher disordered region composition and hydrophobic residue preference than 2,5-HD AICAP. We further compared AICAP with known LLPS prediction tools and revealed that 1,6-HD AICAP values exhibit higher correlation with phase separation probability than 2,5-HD AICAP. Proteins in nuclear condensates, especially self-assembling proteins, are more sensitive to 1,6-HD treatment.

In addition to comparing with these databases, we also compared AICAP with other LLPS experimental methods. FRAP is the standard method for detecting protein mobility and discriminating mobile and immobile protein fraction in condensate. We compared AICAP with FRAP analysis and found that AICAP are associated with protein mobility in cells. Proteins with higher mobility reveal by FRAP (high recovery fraction and low half-time to recover) possess lower AICAP. However, the models and assumptions utilized in FRAP analysis of protein condensates need to consider carefully, especially in living cells [47]. For example, using chemical interaction model, *t*_1/2_ is not completely consistent with AICAP value in our study. Moreover, in the FRAP experiment, protein overexpression brought about changes in protein abundance, protein to RNA ratio and many other factors, resulting in changes in protein aggregation state, and great variation in protein dynamics in FRAP analysis. Considering the complexity in conducting in vitro and in cell FRAP experiments, AICAP may have advantages in testing protein dynamics under endogenous states and cellular concentrations. For example, it was previously reported that heterochromatin binding protein HP1a undergoes phase separation [6]. However, we show here that HP1a exhibits a higher AICAP value. Moreover, Strom et al. also reported that heterochromatin may be initially formed via LLPS, but it gradually matures into an immobile structure no longer sensitive to 1,6-HD treatment [6]. This liquid-solid transition may be necessary for heterochromatin to inhibit transposon activity and maintain the structural stability of the genome.

Integrating Hi-MS and Hi-C data, we found that the dynamic regulatory components, both protein (trans) and DNA (cis), are all more sensitive to 1,6-HD. Recently, an article conducting Hi-C experiment after treating cells with 1,6-HD was published [27]. This article also found that A compartment and E-P loop changed more after 1,6-HD processing, which was consistent with our findings. We further revealed that proteins in nuclear speckles were more sensitive to 1,6-HD compared with nuclear lamina. Another report also found that the transcription active region was more affected by using liquid chromatin Hi-C [45], which further support our findings.

In this study, we enriched genome associated proteins using our newly developed Hi-MS method. We also obtained AICAP values for thousands of proteins and revealed that they are closely related to the chromatin organization stability. However, histone modification also plays critical roles in chromatin condensation [48, 49]. Because histones are tightly bound to DNA, it is difficult for Hi-MS to determine how different modifications change the local condensation. Future studies should target marker proteins/modifications directly using specific antibody, thus target specific condensate in the cytoplasm or nucleus, and find key factors for driving the separation in each type of condensate.

## Method details

### Hexanediol treatment

For isotonic condition, 1,6-Hexanediol (Sigma Cat#240117) was dissolved in RPMI 1640 medium containing 10% FBS to a concentration of 10% to make a storage solution. The working solution was made by dilution using RPMI 1640 medium containing 10% FBS immediately before use. For hypotonic condition, 1,6-Hexanediol was dissolved in H_2_O to a concentration of 30% to make a storage solution. The working solution was made by 1:2 mix of 30% storage solution and RPMI 1640 medium containing 10% FBS immediately before use. 2,5-Hexanediol (Sigma Cat# H11904) was used in the same dosage and condition.

### Hi-MS (chromatin-associated protein capture)

The Hi-MS sample was prepared based on BL-Hi-C protocol to extract chromatin-associated proteins. 10^7^ K562 cells were incubated with 1% formaldehyde in PBS to crosslink protein-DNA in the cells; then, the cells were suspended using 1% SDS lysis buffer (50 mM HEPES-KOH, 150 mM NaCl, 1 mM EDTA, 1% Triton X-100, and 1% SDS). After washing cells with cutsmart buffer with 1%TX-100, the genome was then digested by HaeIII (NEB) into fragments with blunt-ends. The DNA fragments were treated with adenine and ligated with bridge linker with biotin for 4 h at RT. Then, the cells were washed by 0.2% SDS nucleus lysis buffer (20 mM Tris-HCl, 50 mM NaCl, 2 mM EDTA, 0.2% SDS, 1× protease inhibitor) once, then incubate in 0.2% SDS nucleus lysis buffer at 4 °C overnight. The next morning, the cells were washed once again and resuspended in 0.2% SDS nucleus lysis buffer. Cells were sonicated using Digital Sonifier Cell Disruptor at 40% output for 24 cycles, each 5 s on and 5 s off. After sonication, 2× volumes of IP dilution buffer (20 mM Tris pH 8, 2 mM EDTA, 450 mM NaCl, 2% Triton X-100, protease inhibitors) was added and incubate for 1 h at 4°C with rotation. The biotinylated linker/DNA/protein complex in supernatant was then incubated with 1 ml M280 magnet beads slurry (Thermo Fisher Cat#60210) for 2 h at 4 °C with rotation. Beads were then washed 3 times with cold IP wash buffer 1 (20 mM Tris pH 8, 2 mM EDTA, 50 mM NaCl, 1% Triton X-100, 0.1% SDS), once with cold TE buffer (1 mM Tris pH 8, 1 mM EDTA). The complex were eluted twice for 5 min at 100 °C in 60 μl H_2_O each time and sent for label-free quantitative mass spectrometry (MS) analysis.

### Hi-MS (protein sample preparation for mass spec analysis)

#### In-gel digestion of proteins (condition 1)

The gel bands containing the protein sample were manually excised. Each of the protein bands was then digested individually as below. The protein bands were cut into small plugs, washed twice in 200 μl of distilled water for 10 min each time. The gel bands were dehydrated in 100% acetonitrile for 10 min and dried in a Speedvac for approximately 15 min. Reduction (10 mM DTT in 25 mM NH_4_HCO_3_ for 45 min at 56 °C) and alkylation (40 mM iodoacetamide in 25 mM NH_4_HCO_3_ for 45 min at room temperature in the dark) were performed, followed by washing of the gel plugs with 50% acetonitrile in 25 mM ammonium bicarbonate twice. The gel plugs were then dried using a speedvac and digested with sequence-grade modified trypsin (40 ng for each band) in 25 mM NH_4_HCO_3_ overnight at 37 °C. The enzymatic reaction was stopped by adding formic acid to a 1% final concentration. The solution was then transferred to a sample vial for LC-MS/MS analysis.

#### LC-MS/MS analysis (condition 1)

All nanoLC-MS/MS experiments were performed on a Q Exactive (Thermo Scientific) equipped with an Easy-nLC 1000 HPLC system (Thermo Scientific). The peptides were loaded onto a 100-μm id × 2 cm fused silica trap column packed in-house with reversed phase silica (Reprosil-Pur C18 AQ, 5 μm, Dr. Maisch GmbH) and then separated on an a 75-μm id × 20 cm C18 column packed with reversed phase silica (Reprosil-Pur C18 AQ, 3 μm, Dr. Maisch GmbH). The peptides bounded on the column were eluted with a 78-min linear gradient. The solvent A consisted of 0.1% formic acid in water solution and the solvent B consisted of 0.1% formic acid in acetonitrile solution. The segmented gradient was 4–8% B, 8 min; 8–22% B, 50 min; 22–32% B, 12 min; 32–90% B, 1 min; and 90% B, 7 min at a flow rate of 300 nl/min.

The MS analysis was performed with Q Exactive mass spectrometer (Thermo Scientific). With the data-dependent acquisition mode, the MS data were acquired at a high resolution 70,000 (m/z 200) across the mass range of 300–1600 m/z. The target value was 3.00E+ 06 with a maximum injection time of 60 ms. The top 20 precursor ions were selected from each MS full scan with isolation width of 2 m/z for fragmentation in the HCD collision cell with normalized collision energy of 27%. Subsequently, MS/MS spectra were acquired at resolution 17,500 at m/z 200. The target value was 5.00E+ 04 with a maximum injection time of 80 ms. The dynamic exclusion time was 40 s. For nano-electrospray ion source setting, the spray voltage was 2.0 kV; the heated capillary temperature was 320 °C.

#### In-solution digestion of proteins (condition 2)

Protein concentration was determined by Bradford protein assay. Extracts from each sample (40 μg protein) was reduced with 10 mM dithiothreitol at 56 °C for 30 min and alkylated with 10 mM iodoacetamide at room temperature in the dark for additional 30 min. Samples were then digested using the filter-aided sample preparation (FASP) method with trypsin [50]; tryptic peptides were separated in a home-made reverse-phase C18 column in a pipet tip. Peptides were eluted and separated into nine fractions using a stepwise gradient of increasing acetonitrile (6%, 9%, 12%, 15%, 18%, 21%, 25%, 30%, and 35%) at pH 10. The nine fractions were combined to six fractions, dried in a vacuum concentrator (Thermo Scientific), and then analyzed by liquid chromatography tandem mass spectrometry (LC-MS/MS).

#### LC-MS/MS analysis (condition 2)

Samples were analyzed on Orbitrap Fusion Lumos Plus mass spectrometers (Thermo Fisher Scientific, Rockford, IL, USA) coupled with an Easy-nLC 1000 nanoflow LC system (Thermo Fisher Scientific). Dried peptide samples were re-dissolved in solvent A (0.1% formic acid in water) and loaded to a trap column (100 μm × 2 cm, home-made; particle size, 3 μm; pore size, 120 Å; SunChrom, USA) with a max pressure of 280 bar using solvent A, then separated on a home-made 150 μm × 12 cm silica microcolumn (particle size, 1.9 μm; pore size, 120 Å; SunChrom, USA) with a gradient of 5–35% mobile phase B (acetonitrile and 0.1% formic acid) at a flow rate of 600 nl/min for 75 min. For detection with Fusion Lumos mass spectrometry, a precursor scan was carried out in the Orbitrap by scanning m/z 300−1400 with a resolution of 120,000 at 200 m/z. The most intense ions selected under top-speed mode were isolated in Quadrupole with a 1.6 m/z window and fragmented by higher energy collisional dissociation (HCD) with normalized collision energy of 35%, then measured in the linear ion trap using the rapid ion trap scan rate. Automatic gain control targets were 5 × 10^5^ ions with a max injection time of 50 ms for full scans and 5 × 10^3^ with 35 ms for MS/MS scans. Dynamic exclusion time was set as 18 s. Data were acquired using the Xcalibur software (Thermo Scientific).

The mass spectrometry proteomics data have been deposited to the ProteomeXchange Consortium via the PRIDE partner repository [51] with the dataset identifier PXD021434.

#### Data processing and protein quantification

All the MS data were processed in the Firmiana database (Feng et al., 2017). Raw files were searched against the human National Center for Biotechnology Information (NCBI) Refseq protein database (updated on July 4, 2013, 32015 entries) by Mascot 2.3 (Matrix Science Inc.). The mass tolerances were 20 ppm for precursor and 0.05 or 0.5 Da for product ions for Q Exactive (experiment 1) and Fusion (experiment 2), respectively. Up to two missed cleavages were allowed. The data were also searched against a decoy database so that peptide identifications were accepted at a false discovery rate (FDR) of 1%. Proteins with at least 1 unique peptide with Mascot ion score greater than 20 or 2 peptides with Mascot ion score greater than 20 were remained. Label-free protein quantifications were calculated using a label-free, intensity-based absolute quantification (iBAQ) approach (Schwanhausser et al., 2011). The fraction of total (FOT) was used to represent the normalized abundance of a particular protein/peptide across control and treated samples. FOT of protein was defined as a protein’s iBAQ divided by the total iBAQ of all identified proteins within one sample. The FOT was multiplied by 10^6^ for the ease of presentation. The missing data were imputed with the minimum values. After missing value imputation, quantile normalization was applied.

### Statistical analysis

iBAQ values were used in the comparison between control and mock samples and FOT were used in the comparison between control and treated samples. *P*-value was calculated to measure the statistical significance of protein abundance difference of each identified protein in the replicate experiments by *t* test (Supplementary Data 1).

### Immunofluorescence

Coverslips were coated at RT with 5 μg/mL poly-L-lysine solution (Sigma-Aldrich, P4707) for 30 minutes. K562 Cells were plated on the pre-coated coverslips and grown for 1 h followed by fixation using 4% paraformaldehyde (Sigma-Aldrich, 47608) in PBS for 10 min. Then, the cells were permeabilized using 0.5% Triton X-100 (Sigma-Aldrich, X100) in PBS for 10 min. Cells were blocked with 4% Bovine Serum Albumin, BSA (VWR, 102643-516), for 1 h and the indicated primary antibody was added at suitable concentration in PBST for 1 h. Primary antibody used in this study including: MED1 (1:500), Abcam ab64965; FUS (1:500), Sigma-Aldrich HPA008784; EZH2 (1:20), Thermo Fisher MA5-18108; H3K4Me3 (1:2000), Abcam ab8580; RNAPII (1:500), Abcam ab817; and BRD4 (1:500), BETHYL A301-985A50. Cells were washed with PBS three times followed by incubation with secondary antibody at a concentration of 1:1000 in PBS for 1 h. After washing twice with PBS, cells were washed once in water followed by mounting the coverslip onto glass slides with Vectashield (VWR, 101098-042) and finally sealing the coverslip with nail polish (Electron Microscopy Science Nm, 72180). Images were acquired with a Zeiss LSM 880 confocal microscope with 63 × objective using ZEN acquisition software. Images were post-processed using Fiji Is Just ImageJ (FIJI).

### Protein sequence analysis and LLPS annotations

IDR and LCD were predicted using ESpritz (X-ray) and SEG predictor. LLPS prediction was conducted using FuzDrop, PScore, PLAAC, and catGRANULE using default parameters [29]. We classified known LLPS proteins into self-assembling and partner-dependent proteins. The difference is that self-assembling proteins form LLPS droplets alone in vitro while partner-dependent ones undergo LLPS with other components or are recruited into existing condensates.

### Gene ontology over-representation analysis

The over-representation analysis was conducted using R package clusterProfiler v3.18.1 [52].

### FRAP assay and analysis in live cells

The target protein-fluorescent protein Phusion expression vectors were gifts from Xiaohua Shen (PBCAG-WDR43-GFP, PBCAG-dfDDX5-GFP, PBCAG-PSPC1-mCherry, PBCAG-bfH2B-mCherry) and Pilong Li (pCDNA3.1-FUSN-GFP, pCDNA3.1-RNAPIICTD-mCherry, pCDNA3.1-RBM14LCD-GFP), Tsinghua University. The plasmids were transfected into HeLa cell using lipofectamine 3000 reagent and cultured for more than 48 h. Then, the FRAP analysis was performed under on the Zeiss LSM 880 Airyscan following the standard protocol [16]. Briefly, a bleached region, a control non-bleached region, and a control region outside of the cells were selected for each test. After taking 3 baseline images, bleaching of the fluorescence signal was carried out using the corresponding laser (488 nm for GFP and 561 nm for mCherry) at maximum strength. Afterward, an image was taken immediately and then every 1 s for more than 100 s in total. Analysis was carried out by first normalizing the intensity of each bleached spot’s average intensity to average intensity of non-bleached spot at each time point. Next, each time point was converted to a proportion of the original intensity before bleaching. For each experiment, time points after bleaching were fitted to an exponential rise curve with the formula FRAP(*t*) = *A*(1 − *e*^*−t*/*τ*^) + *y*0, where FRAP(*t*) is the fluorescent intensity at time *t* after photobleaching, *A* is the amplitude, *τ* is the time constant, *y*0 is the value of intensity at the first post-bleached frame, and *t* is the time after photobleaching. The time constant and amplitude were optimized to fit the curve using curve_fit function in Python scipy.optimize package. FRAP curve was plotted with a 68% confidence interval (standard error).

### Hi-C

The BL-Hi-C library construction was performed as previously described with some modifications [22]. 10^6^ K562 cells were incubated with 1% formaldehyde in PBS to crosslink protein-DNA in the cells; then, the cells were suspended using lysis buffer (50 mM HEPES-KOH, 150 mM NaCl, 1 mM EDTA, 1% Triton X-100, and 0.1% SDS). The genome was then digested by *Hae*III (NEB) into fragments with blunt-ends. The DNA fragments were treated with adenine and ligated with bridge linker with biotin for 4 h at RT. The unligated DNA fragments were digested with exonuclease (NEB). Next, the cells were digested by Proteinase K (Ambion) overnight, and the DNA was purified using phenol-chloroform (Solarbio) extraction with ethanol precipitation. Then, the ligated DNA was fragmented into 300 bp using an S220 Focused-ultrasonicator (Covaris), and the biotin-labeled DNA fragments were enriched by Dynabeads M280 beads (Thermo Fisher). The enriched DNA library was amplificated by PCR using Q5 DNA polymerase (NEB). After size-selection with the AMPure XP beads (Beckman, Germany), the libraries were sequenced on an Illumina HiSeq X Ten sequencer.

### Data processing

Bridge linkers were trimmed using ChIA-PET2 software [53] using parameters “-A ACGCGATATCTTATC -B AGTCAGATAAGATAT -k 2 -m 1 -e 1”. The resulting clean paired-end reads were aligned independently to hg19 human genome using bwa mem and then processed by HiC-Pro software [54] to obtain valid interaction pairs (“.validPairs”) and subsequent matrix of different resolution. “.hic” files of two conditions were converted from “.validPairs” using Juicer Tools 1.13.02 and used for subsequent analysis.

### Compartment calling

A/B compartment were identified by eigenvector decomposition on the Pearson’s correlation matrix of KR-balanced OE (observed/expected) cis-interaction matrix at 100 kb resolution. The positive and negative values of first eigenvector (PC1) for each 100 kb bin were assigned to A(active) and B(inactive) compartments based on its association with gene density. PC1 ratio = (Hex + PC1 value)/(Hex − PC1 value). The positive value represented that the PC1 value increased after 1,6-hexanediol treatment, suggesting the A/B compartment feature became strengthened. In opposite, the negative value indicated the A/B compartment feature became weakened. We took the change within ± 20% as stable A/B compartment and those beyond ± 20% as weakened/strengthened. Compartments with different signs before and after treatment were annotated as flipped compartments.

### Subcompartment annotation

Rao et al. divided the A/B compartment into five subcompartments namely A1, A2, B1, B2, and B3 based on the regions’ inter-chromosome Hi-C interaction in GM12878 cells, which required as high as 1 kb resolution. Different genomic and epigenetic features were observed in different subcompartments. Xiong et al. developed SNIPER to accurately infer subcompartments based on Hi-C data of moderate depth (~ 500 million mapped reads). Here, we utilized the SNIPER annotation of subcompartment in K562 cells [55].

### Topological associated domain boundary

TAD boundaries were identified using KR-balanced matrix at 40-kb resolution by a Perl script matrix2insulation.pl (https://github.com/dekkerlab/crane-nature-2015) as previously described [56]. The insulation scores were calculated for each chromosome bin by a sliding 1 Mb × 1 Mb square along the diagonal of the matrix. A 200-kb window was used for calculation of the delta vector. TAD boundaries with “Boundary strength” under 0.1 were filtered. TAD boundaries whose centers located within ± 80 kb (2 bins) in two conditions were defined as unchanged boundary.

### Loop calling

Loops were called using HICCUPS [44] at 5/10/20-kb resolution with parameters “-k KR -f 0.1,0.1,0.1 -p 4,2,1 -i 7,5,3 -t 0.02,1.5,1.75,2 -d 20000,20000,50000”. Loops detection before and after treatment were conducted separately and differential loops were annotated as loops that were not detected after treatment using bedtools pairToPair (loops anchor were slopped with 10 kb to avoid false positive).

Aggregation peak analysis (APA) was generated at 5 kb using Juicer APA subcommand with slight modification. Loops were grouped based their subcompartments and the resulted APA matrix were divided by their corresponding number of loops.

Loop signal change was defined as normalized Hi-C contact probability ratio at loop pixels.

### Stripe calling

Stripes were identified using the R script provided by Aleksandra et al. as previous described [46]. The analyses were performed using raw interaction matrices and the normalized matrices generated using juicer software (the .hic files). The matrices were exported to a .txt format from the .hic files using the dump function of juicer. The stripe calling was implemented and performed in R using custom functions.

### RNA-seq

Total RNA was extracted from the K562 cells using TRIZOL (Ambion, USA). The library construction and sequencing were performed by ANOROAD (China).

#### Data processing

Reads were aligned to hg19 genome using hisat2 and resulted sam files were sorted using samtools. The expression profiles were generated using cufflinks cuffnorm with geometric normalization. Signal tracks were produced by deeptools bamCoverage command.

### ATAC-seq

The ATAC library was prepared using Omni-ATAC protocol as previously described. Briefly, 50,000 cells were pellet and resuspend using 50 μl cold ATAC-resuspension buffer (RSB) (10 mM Tris-HCl, 10 mM NaCl, 3 mM MgCl, pH 7.4) containing 0.1% NP40, 0.1% Tween-20, and pipette up and down 3 times. Incubate on ice for 3 min. Wash out lysis with 1 ml of cold ATAC-RSB containing 0.1% Tween-20 but NO NP40 and invert tube 3 times to mix. Pellet and resuspend cells in 50 μl of transposition mixture by pipetting up and down 6 times. The nuclei were then incubated with the Tn5 transposition mix (10 μl 5x TTBL buffer, 3 μl TTE Mix V50 transposase, 16.5 μl PBS, 0.5 μl 10% Tween-20, 20 μl H_2_O) at 37 °C for 30 min (TruePrep® DNA Library Prep Kit V2 for Illumina, Vazyme, China). After the tagmentation, the stop buffer was directly added to the reaction to end the tagmentation. PCR was performed to amplify the library in 12 cycles. After the PCR reaction, the libraries were purified with 1.2× AMPure beads (Beckman, Germany). The libraries were sequenced using an Illumina HiSeq X Ten sequencer.

ATAC-seq raw reads were trimmed to remove adaptor sequence and mapped to hg19 genome with Bowtie2 using parameters “--very-sensitive -X 2000” and duplicates were removed using Picard MarkDuplicates command. Signal tracks were produced by deeptools bamCoverage command. Peaks were called by MACS2 using parameters “--nomodel --shift -100 --extsize 200 -B --call-summits –SPMR”.

### Restriction endonuclease recognition motif frequency

Recognition motif GGCC (HaeIII) and GATC (DpnII and HindIII) frequency were defined as occurrence times per 500 bp across genome, which was transform to bigwig coverage by deeptools bamCoverage. The signal at H3K27ac/EZH2 ChIP-seq peaks were generated using deeptools computeMatrix.

## Supplementary Information

**Additional file 1: Supplementary figures**

**Additional file 2: Supplementary Table S1**. Table S1. List of AICAP values for Hi-MS captured chromatin-associated proteins. The list contains 1,6-HD and 2,5-HD treatment MS data. 1,6-HD treatment MS data were prepared using 2 conditions (sheet 1,6-HD-1 and 1,6-HD-2). More proteins were obtained using condition 2, so the MS data used in all figures were form condition 2. 2,5-HD treatment experiment was conducted under condition2.

**Additional file 3: Supplementary Table S2**. Table S2: List of LLPS related annotation used in this paper.

**Additional file 4: Supplementary Table S3**. Table S3. Quality control of BL-Hi-C experiments.

**Additional file 5: Supplementary Table S4**. Table S4. List of matched GO terms of all proteins captured by Hi-MS.

**Additional file 6.** Review history.

## Data Availability

BL-Hi-C, RNA-seq, and ATAC-seq data are available on Sequence Read Archive under project accession PRJNA645615 [57]. The mass spectrometry proteomics data have been deposited to the ProteomeXchange Consortium via the PRIDE partner repository with the dataset identifier PXD021434 [58] and PXD027565 [59]. Previously published ChIP-seq data used in this paper can be found at Gene Expression Omnibus under accession GSE117492 [16] or at ENCODE database [60] under accession ENCFF359UWD, ENCFF487UYG, and ENCFF384ZZM (https://www.encodeproject.org/).
