# Supplementary material for: Quantifying the phase separation property of chromatin-associated proteins under physiological conditions using an anti-1,6-hexanediol index
Source: Genome Biol. 2021 Aug 17;22:229. doi: 10.1186/s13059-021-02456-2 (PMC8369651; doi:10.1186/s13059-021-02456-2)
Supplement: Supplementary file 1 — Additional file 1: Supplementary figures [file 13059_2021_2456_MOESM1_ESM.pdf]

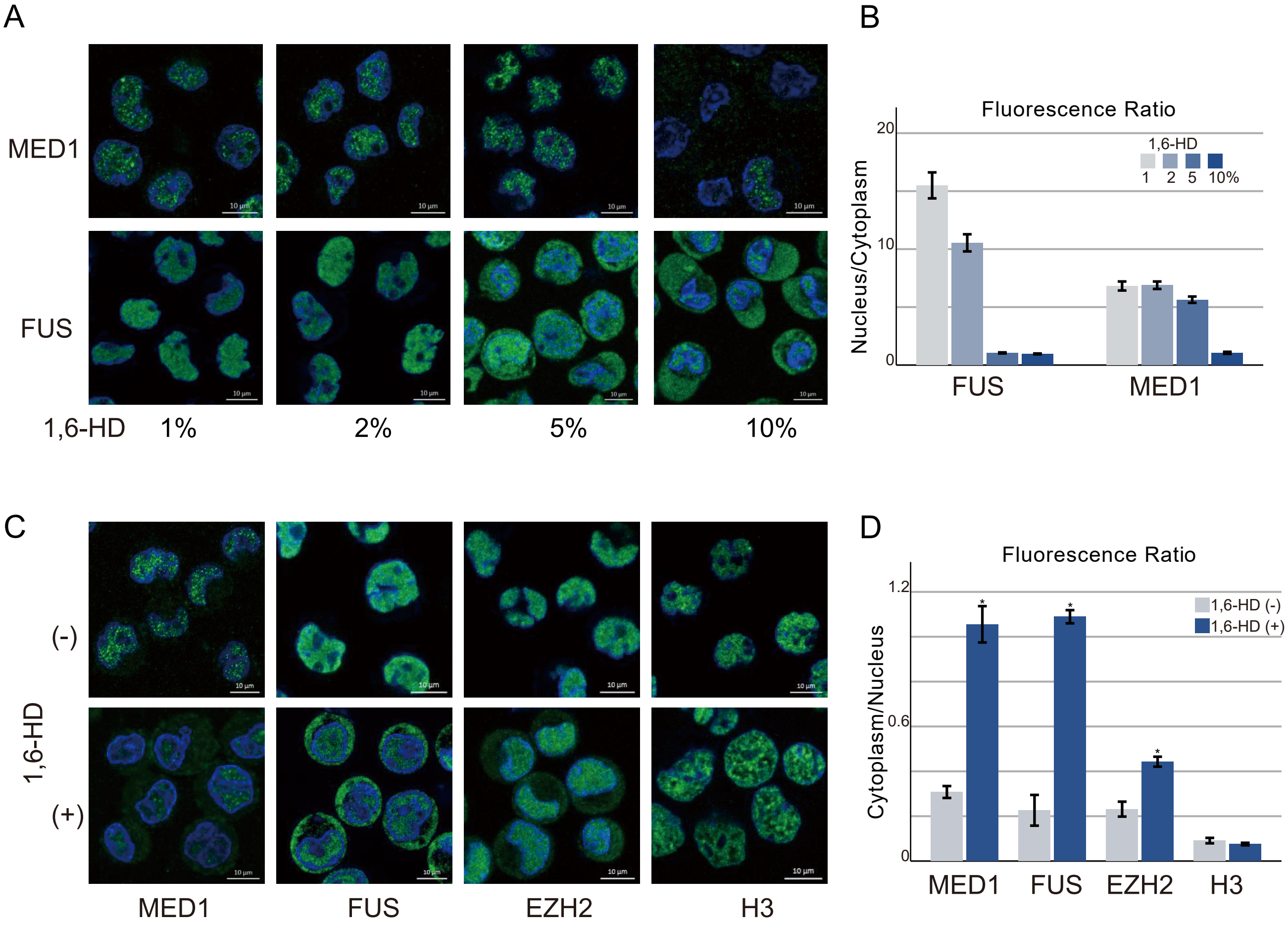

Figure S1. Proteins exhibit different sensitivities to 1,6-HD treatment.
A. MED1/FUS distribution at 1,6-HD concentration from 1-10% in K562 cells. Incubation time: 20min. Blue: DAPI, green: MED1/FUS. Scale bar: 10μm. **B.** Quantification of nuclear/cytoplasmic fluorescence signal for MED1/FUS upon 1,6-HD treatment (n = 100), as in (A). **C.** Nuclear-cytoplasmic distribution of MED1, FUS, EZH2 and H3K4me3 after 10% 1,6-HD treatment for 20 mins in hypotonic condition. **D.** Quantification of cytoplasmic/nuclear fluorescence signal for MED1, FUS, EZH2 and H3K4me3 upon 1,6-HD treatment (n = 100), as in (B).

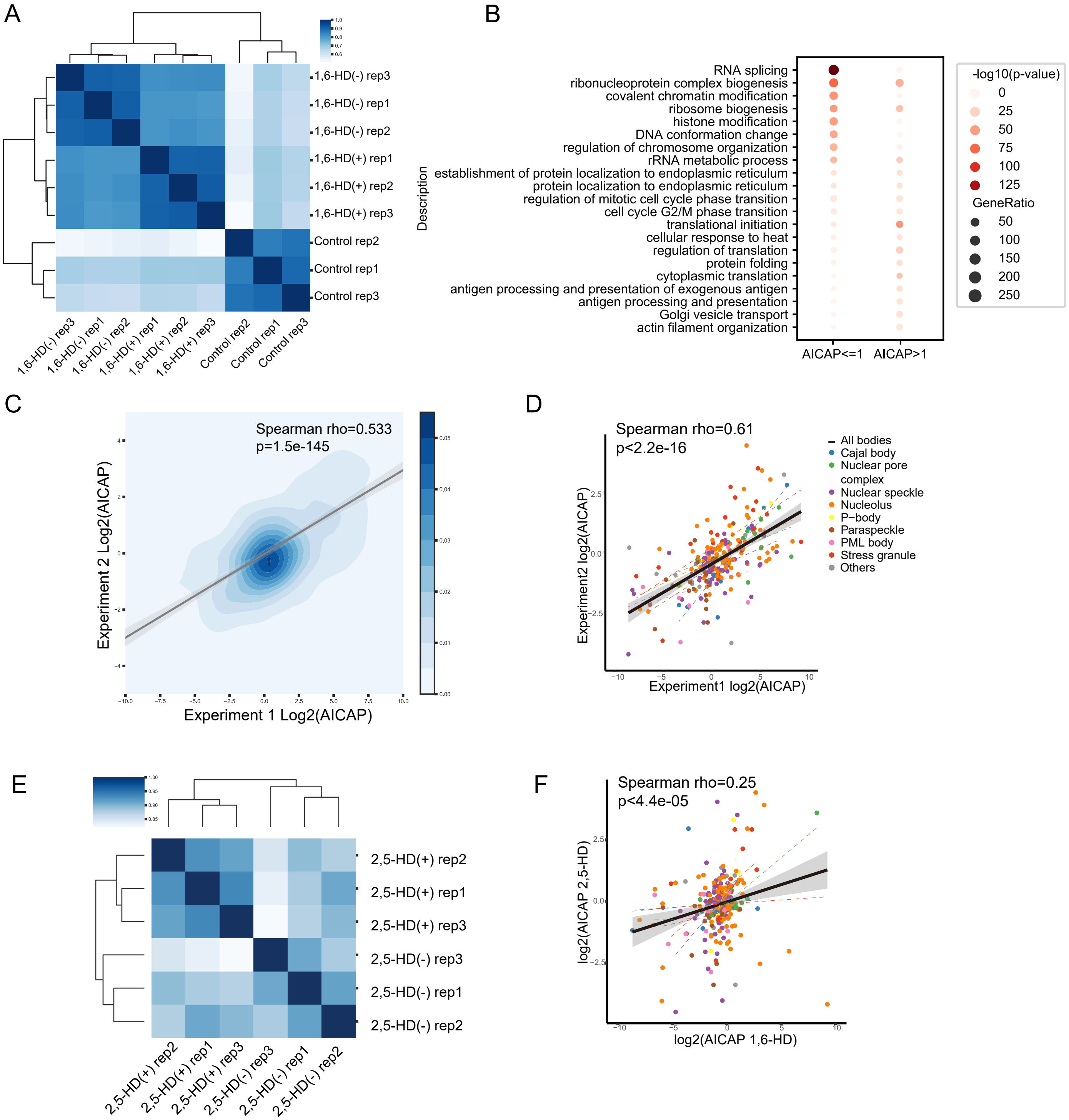

Figure S2. Reproducibility of Hi-MS and AICAP.

A. Clustering of replicated Hi-MS samples using Spearman correlation of protein content (iBAQ signal). (-): wild type samples, (+): 1,6-HD treated samples and control: undigested control samples. **B.** Functional enrichment of proteins with AICAP values ≤1 or > 1. **C.** Spearman correlation of AICAP values of two independent experiments. They were prepared from different batches of cells, different types of digestion (in gel or in solution) and different types of mass spectrometers. **D.** Correlation of AICAP values of proteins in nuclear domains between two independent replicates. **E.** Clustering of replicated 2,5-HD treatment Hi-MS samples using Spearman correlation of protein content (iBAQ signal). **F**. Correlation of AICAP values of proteins in nuclear domains between 1,6-HD and 2,5-HD treatment samples.

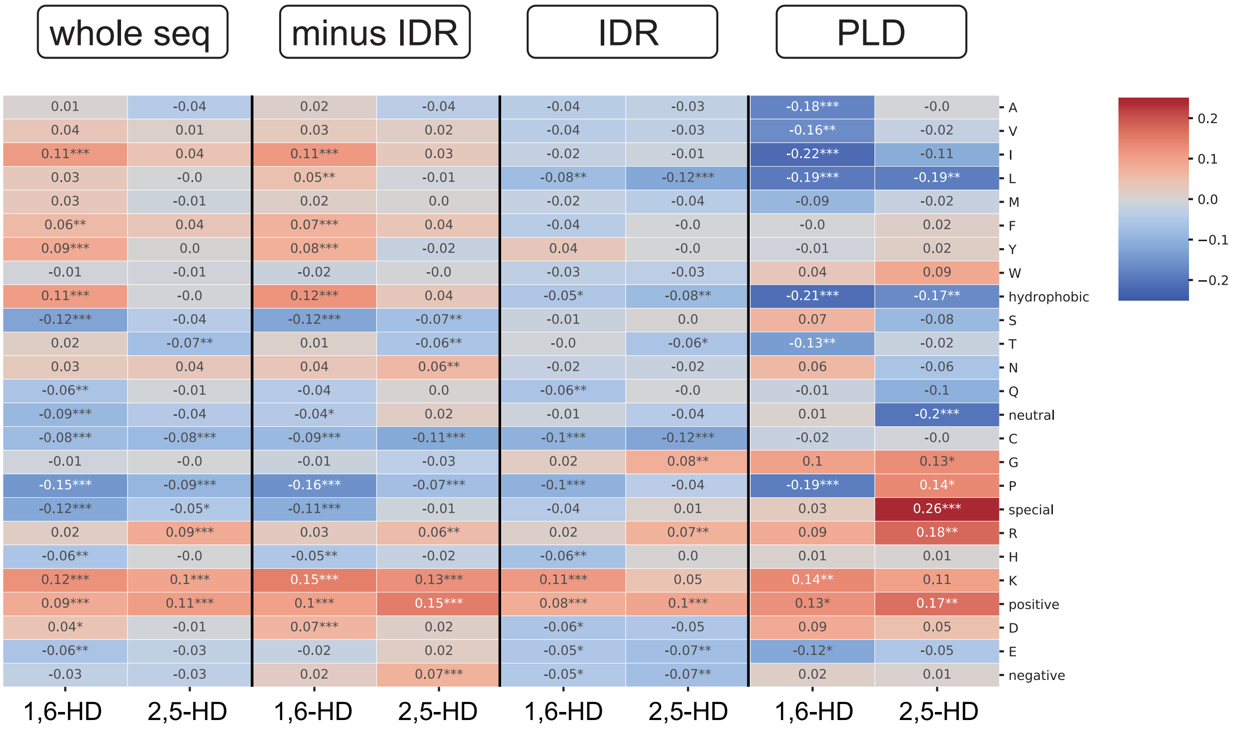

Figure S3. Correlation between AICAP and protein residue composition.

The Spearman correlation between AICAP and corresponding residue percentage in proteins with AICAP < 1. Each column panel refers to residue percentage in different regions(whole sequence, whole sequence without IDR, IDR and PLD). ***: p-value < 0.01, **: p-value < 0.05, * p-value < 0.1.

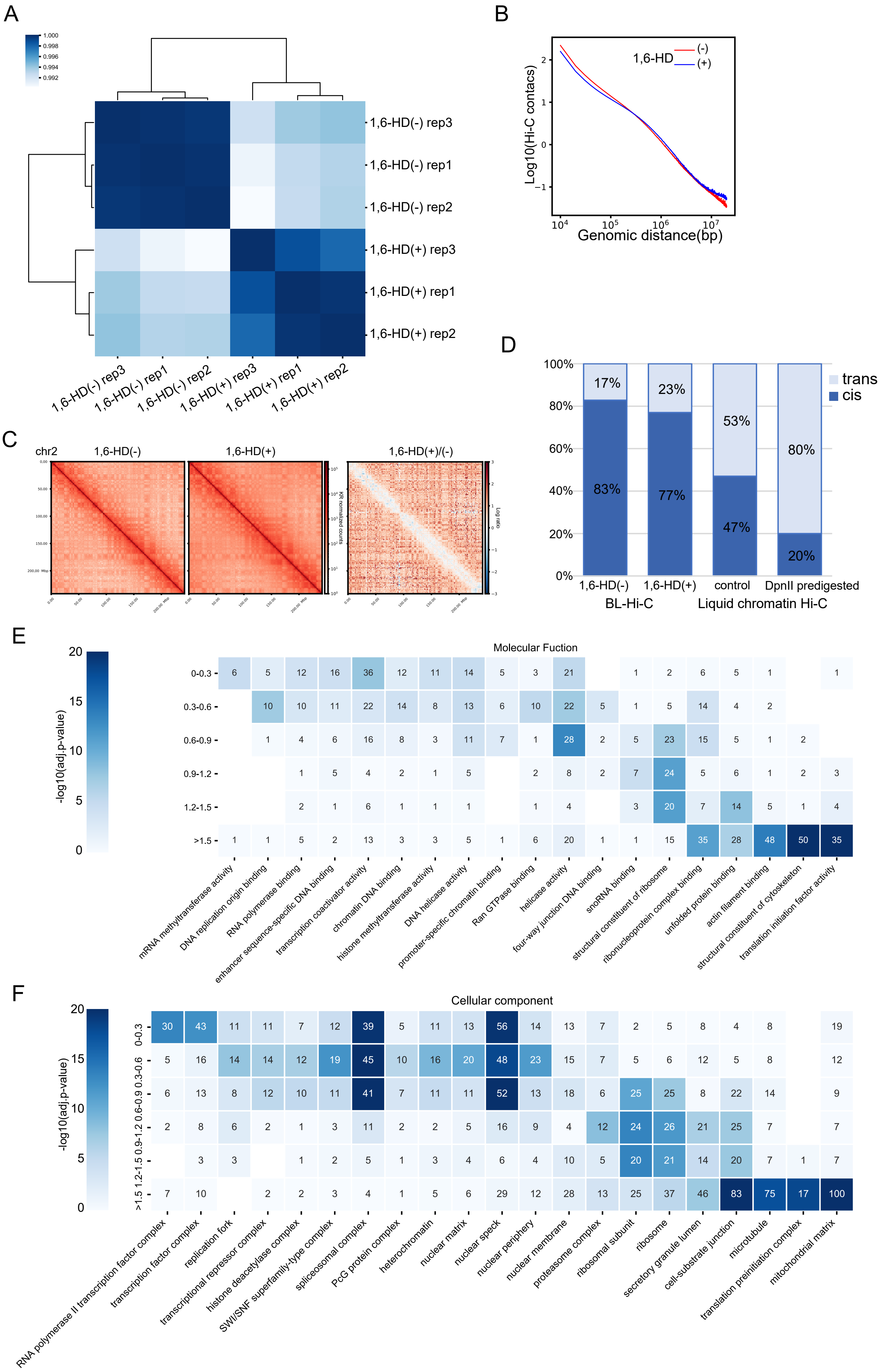
A
1,6-HD(-) rep3
1,6-HD(-) rep1
1,6-HD(-) rep2
1,6-HD(+) rep3
1,6-HD(+) rep1
1,6-HD(+) rep2
B
1,6-HD
(-)
(+)
Log10(Hi-C contacs)
Genomic distance(bp)
C
chr2
1,6-HD(-)
1,6-HD(+)
1,6-HD(+)/(-)
KR normalized counts
Log ratio
D
trans
cis
17%
83%
23%
77%
53%
47%
80%
20%
1,6-HD(-)
1,6-HD(+)
control
DpnII predigested
BL-Hi-C
Liquid chromatin Hi-C
E
Molecular Fuction
-log10(adj.p-value)
0-0.3
0.3-0.6
0.6-0.9
0.9-1.2
1.2-1.5
>1.5
mRNA methyltransferase activity
DNA replication origin binding
RNA polymerase binding
enhancer sequence-specific DNA binding
transcription coactivator activity
chromatin DNA binding
histone methyltransferase activity
DNA helicase activity
promoter-specific chromatin binding
Ran GTPase binding
helicase activity
four-way junction DNA binding
snoRNA binding
structural constituent of ribosome
ribonucleoprotein complex binding
unfolded protein binding
actin filament binding
structural constituent of cytoskeleton
translation initiation factor activity
F
Cellular component
-log10(adj.p-value)
RNA polymerase II transcription factor complex
transcription factor complex
replication fork
transcriptional repressor complex
histone deacetylase complex
SWI/SNF superfamily-type complex
spliceosomal complex
PcG protein complex
heterochromatin
nuclear matrix
nuclear speck
nuclear periphery
nuclear membrane
proteasome complex
ribosomal subunit
ribosome
secretory granule lumen
cell-substrate junction
microtubule
translation preinitiation complex
mitochondrial matrix

Figure S4. Quality control of BL-Hi-C and gene ontology enrichment analysis of proteins.
A. Clustering of replicated BL-Hi-C samples. **B.** Genome-wide Hi-C interaction frequency changes before (-) and after (+)1,6-HD treatment. **C.** BL-Hi-C interaction matrices for chr2 at 1Mb resolution. **D.** Data quality of BL-Hi-C and liquid chromatin Hi-C in K562 cells (Belaghzal et al., 2019). Cis: intra-chromosomal interaction, trans: inter-chromosomal interaction. Trans interactions was generally considered to be the noise. **E.** and **F.** Gene ontology molecular function and cellular component enrichment analysis of all captured proteins in each group.

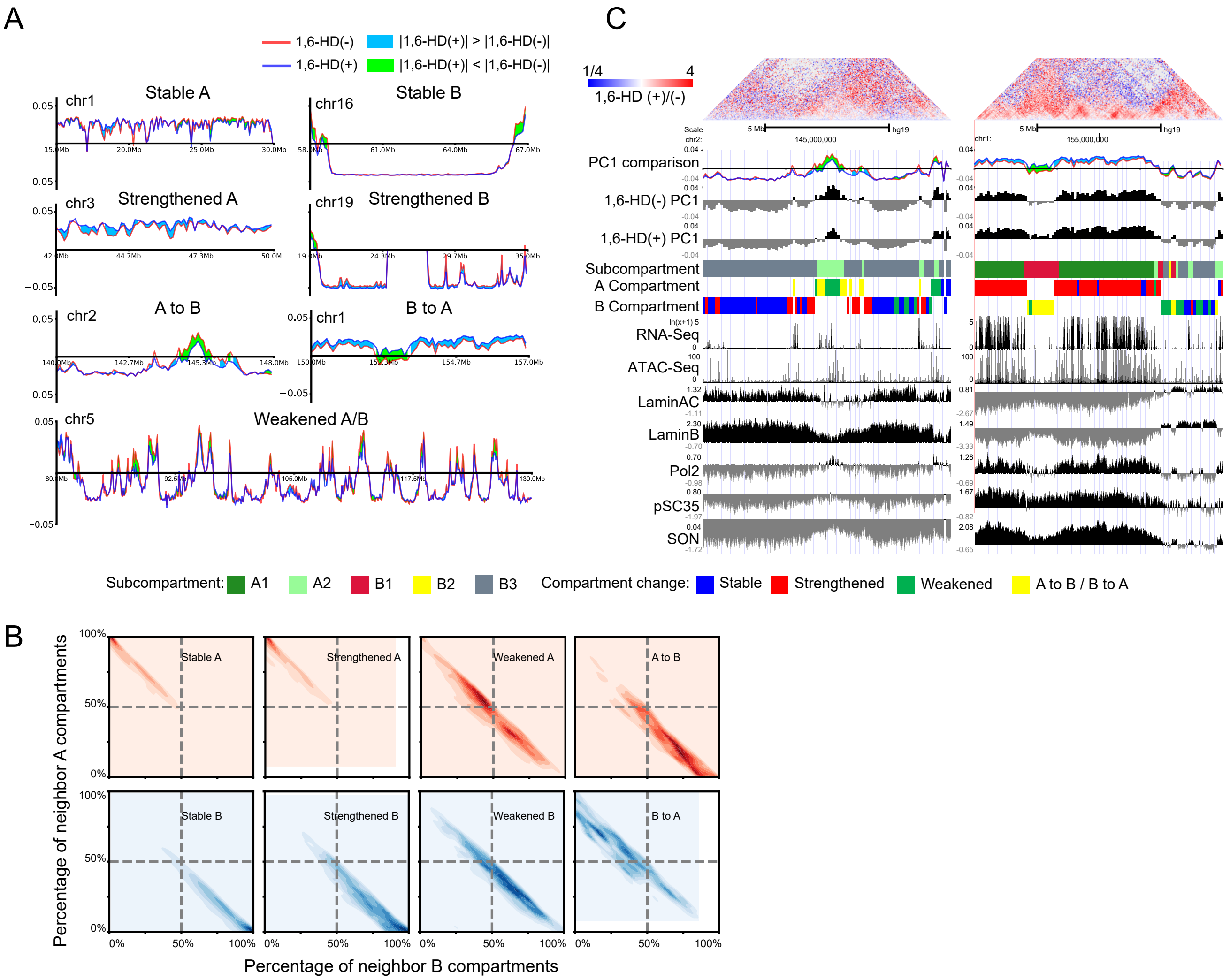

Figure S5. Compartment change-types and neighborhood relationship.
A. Examples of stable, strengthened, weakened and flipped compartments before (-) and after (+)1,6-HD treatment. The y axis shows PC1 values. **B.** Percentage of neighbor compartments of each compartment change-types. Neighborhood was defined as compartments within 3Mb as usual. **C.** Examples of weakened/flipped A(left) and B(right) compartments and corresponding nuclear speckle/lamina TSA-seq[45] plots.

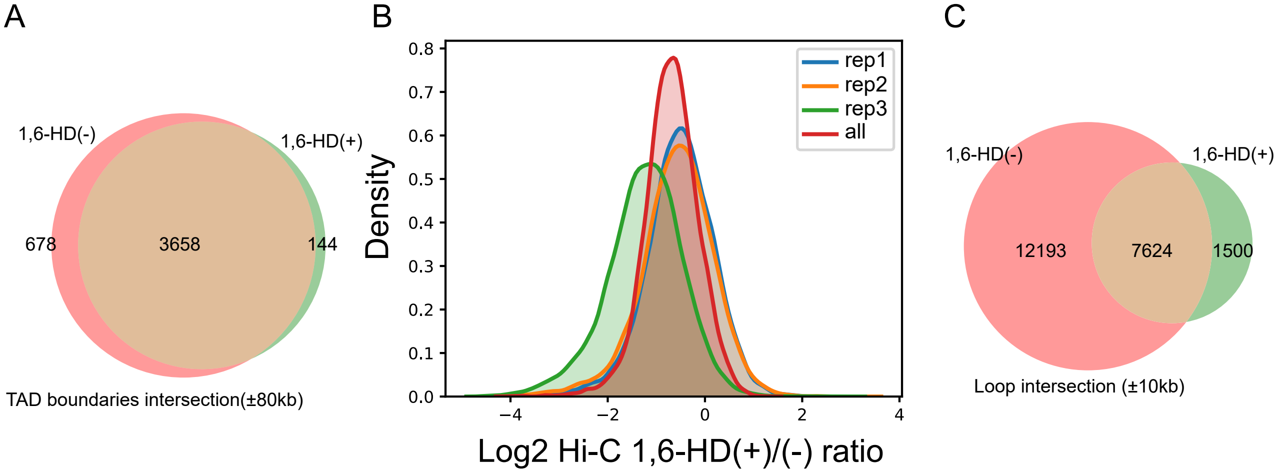

Figure S6. Chromatin TAD and loop changes after 1,6-HD treatment.
A. Identified TAD boundaries before (-)and after (+)1,6-HD treatment. **B.** DNA loop anchor interaction changes after 1,6-HD treatment. **C.** Identified DNA loops before (-) and after (+)1,6-HD treatment. More than 60% of loops can not be identified again after 1,6-HD treatment.
