# Supplementary material for: Quantifying the phase separation property of chromatin-associated proteins under physiological conditions using an anti-1,6-hexanediol index
Source: Genome Biol. 2021 Aug 17;22:229. doi: 10.1186/s13059-021-02456-2 (PMC8369651; doi:10.1186/s13059-021-02456-2)
Supplement: Supplementary file 4 — Additional file 4: Supplementary Table S3. Table S3. Quality control of BL-Hi-C experiments. [file 13059_2021_2456_MOESM4_ESM.pdf]

TableS3 Quality control of BL-Hi-C experiments

Condition	replicate	total PETs	Valid PETs	Unique_paired_alignments	Valid_interaction_pairs	valid_interaction_rmdup	trans_interaction	cis_interaction	cis/trans
1,6-HD(-)	rep1-1	29438000	25331673	16935472	11557498	-	-	-	-
	rep2-1	33252468	28509616	18677317	13467020	-	-	-	-
	rep3-1	45893846	39778132	26418191	19407786	-	-	-	-
	rep1-2	749122163	644608079	428365449	290268716	-	-	-	-
	rep2-2	755428345	647842397	421512295	301757571	-	-	-	-
	rep3-2	776025450	672469129	444162683	324363301	-	-	-	-
	**Summary**	2389160272	2058539026	1356071407	960821892	649465091	111696727	537768364	4.81
1,6-HD(+)	rep1-1	41589365	35909324	22788602	18977150	-	-	-	-
	rep2-1	38072170	31992728	20027819	16345180	-	-	-	-
	rep3-1	29154137	24401207	14952963	11759364	-	-	-	-
	rep1-2	747180413	645103059	405872089	335937700	-	-	-	-
	rep2-2	666975425	558536859	343261747	276368714	-	-	-	-
	rep3-2	586417942	489337783	295982287	229752090	-	-	-	-
	**Summary**	2109389452	1785280960	1102885507	889140198	647066749	148821974	498244775	3.35
