# Supplementary material for: Quantifying the phase separation property of chromatin-associated proteins under physiological conditions using an anti-1,6-hexanediol index
Source: Genome Biol. 2021 Aug 17;22:229. doi: 10.1186/s13059-021-02456-2 (PMC8369651; doi:10.1186/s13059-021-02456-2)
Supplement: Supplementary file 6 — Additional file 6. Review history. [file 13059_2021_2456_MOESM6_ESM.docx]

Review History

**First round of review**

**Reviewer 1**

**Are you able to assess all statistics in the manuscript, including the appropriateness of statistical tests used?** Yes, and I have assessed the statistics in my report.

**Comments to author:**

The authors developed a method, Hi-MS, to detect the set of proteins bound to DNA genome-wide. This method combines chromatin-associated protein pull-down and quantitative proteomics. It uses restriction endonuclease HaeIII to cut at GGCC sites to enrich cis-regulatory elements of gene promoters. Using this method, the authors quantified the changes before and after 1,6-HD treatment and revealed differential sensitivity of protein with respect to the treatment. In addition, they performed Hi-C experiments to characterize the impact of 1,6-HD treatment on chromatin organization. While I agree that the datasets generated are useful, I have concerns about the presentation and data interpretation.

Major comments:

The authors should avoid using LLPS everywhere, because there is no evidence that the changes seen in the experiments result from disrupting LLPS. 1,6-HD is a rather general molecule that disrupts protein-protein and protein-DNA interactions. Its exact impact inside the nucleus is unclear. For example, a recent study by the Maeshima group [PMID: 33536240] shows that "1,6-HD drastically suppresses chromatin motion and hyper-condenses chromatin in human cells by using live-cell single-nucleosome imaging". This non-specific, global compaction will have significant implications on many of the observations shown in the current study.

For example, suppose the change observed in Hi-C is mostly driven by the chromosome compaction upon 1,6-HD treatment and not related to dissolving liquid droplets. In that case, the whole section "Nuclear condensates affect various levels of chromatin organization" should be revised. For example, the sentence "these results strongly indicated that chromatin changes in TADs and loops were related to proximate nuclear condensates" wouldn't be supported by the data at all.

In my opinion, the interpretation of the AICAP value is non-trivial. It's not clear to me that proteins with increase AICAP values would disprove their role in LLPS. For example, it is possible that the proteins are initially sequestered in nuclear condensates not bound to chromatin. Upon disrupting such condensates, protein molecules are released and would, in fact, bind more frequently with chromatin.

Minor comments:

·       The results shown in Figure 3E and 3F are of poor quality. They do not support LLPS. The authors should consider performing experiments, such as fluorescence recovery after photobleaching, to validate the dynamical property of the clusters.

·       This sentence is not really supported by the data "The result showed that most proteins in nuclear domains such as nuclear speckles, paraspeckles and PML bodies exhibit low AICAP values, which is in agreement with a previously published LLPS database[25].". Many proteins in stress granules and nucleolus have larger AICAP values.

·       It is unclear to me the rationale for using NPC to evaluate the efficiency of Hi-MS method in enriching chromatin associated proteins. Why are they enriched much more than TF and DNA-templated transcription?

·       There is a misspelling in Fig 1D legend. "Undogested" should be "Undigested".

**Reviewer 2**

**Are you able to assess all statistics in the manuscript, including the appropriateness of statistical tests used?** No, I do not feel adequately qualified to assess the statistics.

**Comments to author:**

Developing a systematic experimental methodology to identify and characterise biomolecular condensates is crucial for further development of the LLPS field.
The present study aims to achieve this goal by investigating proteins related to organisation and regulation of chromatin.
The approach is based on 'differential' interactions with 1,6 HD, exploiting hydrophobic patches within disordered condensate interactions.

Major points.
- Owing to the immense interest in the field and the concerns on the quality of the results in general, including some false reports, the authors should be super-careful in presenting their method.
Please clearly provide a set of negative examples.
Please relate your results to those in Boija et al (2018, Cell)  an Tjian group (2018, Science). I have the impression that some results are contradictory.

In particular try to define the difference between hubs and condensates (see PMID: 29930090) and compare the different sensitivity to 1,6 HD. The recent debate in Science Jan, 2021
reflects the concerns and the new method should be able to address these concerns.

The 2018 Cell study provides an in-depth view on the interactions within the condensates. One must be careful, the interactions are disordered, but versatile (PMID: 33318217), some have distinguished
hydrophobic patches, whereas others do not. Also depending on the propensity for aggregation. It would be useful to elaborate this point.
As disorder in itself is not a certain measure of disordered interactions , which can also be formed using structured domains, I suggest using some predictions of these interactions on selected positive and negative examples,
and show the direct relationship between high AICAP scores and disordered interactions. Recently, the condensates could be predicted based on this feature, as described by Hahn (cell, 2018), Boija (cell, 2018)

The Discussion does not fit well with the rest of the paper. This is a method paper , which should serve as a general approach to study condensates . I expect the discussion about more the technical aspects, how this compares
to other approaches,  whether biases for aggregation prone proteins etc, and much less about functional conclusions etc.
As condensates are formed by drivers and clients and their assembly/disassembly is regulated by a series of posttranslational modifications, I do not find these conclusions sufficiently founded. The community would benefit from
discussing perspectives on systematic studies rather than making systems biology conclusions on novel, developing methodologies.

Minor point
I suggest the authors to be a bit less technical and give a chance for a wider community to understand the approach and perspectives.
I also suggest to be careful with the examples, and compare to all possible data in literature, as some are controversial.
I think the work can be really valuable, if stays more method focused and discuss all the details related to methodology.

**Authors Response**

**Point-by-point responses to the reviewers’ comments:**

Reviewer #1:

Comment: The authors developed a method, Hi-MS, to detect the set of proteins bound to DNA genome-wide. This method combines chromatin-associated protein pull-down and quantitative proteomics. It uses restriction endonuclease HaeIII to cut at GGCC sites to enrich cis-regulatory elements of gene promoters. Using this method, the authors quantified the changes before and after 1,6-HD treatment and revealed differential sensitivity of protein with respect to the treatment. In addition, they performed Hi-C experiments to characterize the impact of 1,6-HD treatment on chromatin organization. While I agree that the datasets generated are useful, I have concerns about the presentation and data interpretation.

**Response: We thank the reviewer for his/her positive appreciation of our contribution and constructive criticism.**

Major comments:

Comment: The authors should avoid using LLPS everywhere, because there is no evidence that the changes seen in the experiments result from disrupting LLPS. 1,6-HD is a rather general molecule that disrupts protein-protein and protein-DNA interactions. Its exact impact inside the nucleus is unclear. For example, a recent study by the Maeshima group [PMID: 33536240] shows that "1,6-HD drastically suppresses chromatin motion and hyper-condenses chromatin in human cells by using live-cell single-nucleosome imaging". This non-specific, global compaction will have significant implications on many of the observations shown in the current study. For example, suppose the change observed in Hi-C is mostly driven by the chromosome compaction upon 1,6-HD treatment and not related to dissolving liquid droplets. In that case, the whole section "Nuclear condensates affect various levels of chromatin organization" should be revised. For example, the sentence "these results strongly indicated that chromatin changes in TADs and loops were related to proximate nuclear condensates" wouldn't be supported by the data at all.

**Response: We thank the reviewer for the suggestion. We agree that we should avoid using LLPS everywhere. In the revised manuscript, we described the changes after 1,6-HD treatment using AICAP instead of using LLPS.**

**For the influence of chromosome compaction, as demonstrated in the report [PMID: 33536240], both 1,6-HD and 2,5-HD can cause the global compaction. In this revision, we performed the Hi-MS experiments with 2,5-HD treatment. By comparing the response of chromatin associated proteins to 1,6-HD and 2,5-HD treatments, we found that: 1) proteins with low 1,6-HD AICAP values exhibit higher disordered region composition and hydrophobic residue preference than 2,5-HD AICAP (Fig. 2C, D). 2) 1,6-HD AICAP values exhibit higher correlation with phase separation predictors than 2,5-HD AICAP (Fig. 2D). 3) 1,6-HD cause stronger disruption effect against interactions within biomolecular condensates than 2,5-HD, especially liquid condensate and the self-assembling proteins (Fig. 3). These results showed that though both 1,6-HD and 2,5-HD resulted in chromosome compaction, the response of chromatin associated proteins to 1,6-HD treatment related more to their LLPS property.  See pages 6-8 of the revised manuscript for details.**

**Though by comparing 1,6-HD and 2,5-HD, we found that observed AICAP is not non-specific effect of global compaction, we still changed the section title of "Nuclear condensates affect various levels of chromatin organization" into “Active Chromatin TADs/loops are more sensitive to 1,6 HD treatment” and removed the sentence "these results strongly indicated that chromatin changes in TADs and loops were related to proximate nuclear condensates" and related content. Related modifications can be found from pages 11-12.**

Comment: In my opinion, the interpretation of the AICAP value is non-trivial. It's not clear to me that proteins with increase AICAP values would disprove their role in LLPS. For example, it is possible that the proteins are initially sequestered in nuclear condensates not bound to chromatin. Upon disrupting such condensates, protein molecules are released and would, in fact, bind more frequently with chromatin.

**Response: We thank the reviewer for the suggestion. The interpretation of the AICAP value is really non-trivial. We also agree that proteins with increased AICAP values would not disprove their role in LLPS. However, we didn’t make the point clear in the original manuscript.**

**It is possible that contact with DNA will increase after the disruption of condensate, 41% of proteins in nucleolus possess AICAP values >1 (Figure 3A). However, many proteins, such as RPLs and RPSs, distributed in both ribosome and nucleolus. Previous reports have shown that 1,6-HD can dissolve NPC channel and ribosome components, therefore, ribosome components might go into the nucleus and exhibit increased AICAP values. Moreover, besides ribosome proteins, many cytoplasmic proteins involved in protein synthesis, folding, and transport may enter the nucleus after 1,6-HD treatment (Figure X) and exhibit AICAP value>1, this makes it difficult to distinguish the proteins with AICAP>1 whether derived from the destroyed nuclear condensates or from the cytoplasm. Together, though increased AICAP values would not disprove their roles in phase separation, this part of data should be interpreted with caution so we pay more attention to proteins with decreased AICAP values in this study. See Page 7 for details.**

Minor comments

Comment: The results shown in Figure 3E and 3F are of poor quality. They do not support LLPS. The authors should consider performing experiments, such as fluorescence recovery after photobleaching, to validate the dynamical property of the clusters.

**Response: We agree that fluorescence recovery after photobleaching (FRAP) should be used to validate the dynamical property of the clusters. In this revision, we added the FRAP experiments of 7 proteins, which included FUSN, RNAPIICTD, H2B, bfDDX5, WDR43, PSPC1 and RBM14LCD. FRAP results demonstrated that proteins with high mobility displayed low AICAP values. A new section (Page 9) and Figure 4 was added in this revision.**

Comment: This sentence is not really supported by the data "The result showed that most proteins in nuclear domains such as nuclear speckles, paraspeckles and PML bodies exhibit low AICAP values, which is in agreement with a previously published LLPS database[25].". Many proteins in stress granules and nucleolus have larger AICAP values.

**Response: We are sorry for the imprecise sentence and we have re-organized this part in this revision. We divided the biomolecular condensates into two classes. The first class is typical nuclear condensates, including nuclear speckles, paraspeckles and PML bodies, most proteins in this class exhibit low AICAP values. While the other class is condensates closely associated with cytoplasm, including stress granule, nuclear pore complex or ribosomes/nucleolus. Many proteins in this class exhibit high AICAP values, which may go into the nucleus and bind to after NPC disruption. To better display the differences between these two classes, we changed original Fig. 2C to box-plot (Fig. 3A). See page 7 for the rephrased expression and we hope it is clearer.**

Comment: It is unclear to me the rationale for using NPC to evaluate the efficiency of Hi-MS method in enriching chromatin associated proteins. Why are they enriched much more than TF and DNA-templated transcription?

**Response: Thanks for the suggestion. NPC is a well-studied multi-protein complex with components located at both sides of nuclear membrane (Fig. 1E). We consider that NPC components at cytoplasm side (NUP214) would be less associated with chromatin than components at nucleus side (NUP153) or both sides (NUP98). Moreover, another method to capture chromatin-associated proteins at specific genomic locus (Liu et al., 2017, Cell) also confirmed that NPC components at nuclear side was enriched in their captured protein set. Hence, the enrichment of NPC would be a good indicator to reflect the efficiency of Hi-MS to enrich chromatin associated proteins. The result showed that the enrichment ratio of different components was consistent with their cellular location (NUP214 < NUP98 < NUP153).**

**Generally, for proteins of functions mentioned in Figure 1D and NPC, their average protein abundance captured by Hi-MS didn’t show significant difference. However, most NPC components were poorly captured in the control samples, resulting in relative high fold enrichment ratio versus control.**

Comment: There is a misspelling in Fig 1D legend. "Undogested" should be "Undigested".

**Response: We are very sorry for this typo error, and have corrected them accordingly.**

Reviewer #2:

Comment: Developing a systematic experimental methodology to identify and characterise biomolecular condensates is crucial for further development of the LLPS field.

The present study aims to achieve this goal by investigating proteins related to organisation and regulation of chromatin.

The approach is based on 'differential' interactions with 1,6 HD, exploiting hydrophobic patches within disordered condensate interactions.

**Response: We thank the reviewer for the concise summary to our work.**

Major points.

Comment: - Owing to the immense interest in the field and the concerns on the quality of the results in general, including some false reports, the authors should be super-careful in presenting their method.

Please clearly provide a set of negative examples.

**Response: We thank the reviewer for raising this issue. In the revised manuscript, we compare the changes after 1,6-HD treatment to widely used phase separation predictors, LLPS related features such as content of intrinsically disordered regions and FRAP experiments which are regular used for studying protein mobility, instead of simply defined it as LLPS.**

**2,5-HD is often used as a control for 1,6-HD, therefore we provided a complete list of 2,5-HD AICAP by perform Hi-MS using 2,5-HD treatments (Additional file 2). we found that: 1) proteins with low 1,6-HD AICAP values exhibit higher disordered region composition and hydrophobic residue preference than 2,5-HD AICAP (Fig. 2C, D). 2) 1,6-HD AICAP values exhibit higher correlation with phase separation predictors than 2,5-HD AICAP (Fig. 2D). 3) 1,6-HD cause stronger disruption effect against interactions within biomolecular condensates than 2,5-HD, especially liquid condensate and the self-assembling proteins (Fig. 3). Though we cannot simply used 2,5-HD as “negative” control, the comparison between 1,6-HD and 2,5-HD demonstrated that 1,6-HD is the consequence of specific disruption against hydrophobic interactions rather than non-specific global effect. See pages 6-8 of the revised manuscript for details.**

Comment: Please relate your results to those in Boija et al (2018, Cell)  an Tjian group (2018, Science). I have the impression that some results are contradictory.

In particular try to define the difference between hubs and condensates (see PMID: 29930090) and compare the different sensitivity to 1,6 HD. The recent debate in Science Jan, 2021 reflects the concerns and the new method should be able to address these concerns.

**Response: We thanks the reviewer for raising this question for us to think deeply. In the 2018 Science work of Tjian group, EWS, TAF15 and SP1 didn’t display obvious puncta, and they proposed a concept of hubs to describe these transient local high-concentration biomolecules in vivo. The AICAP value of EWS, TAF15 and SP1 was EWS, 0.431 TAF15, 0.525 and Sp1 0.370, respectively.**

**In the 2018 Cell work, Boija et al. focus on condensates in ES cells, and obvious puncta were observed for OCT4, SOX2, NANOG, MYC, P53 and ER. However, our datasets were generated in K562 cells and 1,6-HD AICAP were unavailable for all these six proteins.**

**For the added comparisons in this revision, such as the different sensitivity to 1,6 HD among liquid, gel-like, and solid-state proteins, and the different sensitivity to 1,6 HD between self-assembling and partner-dependent proteins, we compared AICAP with theses classified proteins and address some concerns in this field. However, it is hard to compare the AICAP between hubs and condensates due to the lack of grouping criteria.**

Comment: The 2018 Cell study provides an in-depth view on the interactions within the condensates. One must be careful, the interactions are disordered, but versatile (PMID: 33318217), some have distinguished hydrophobic patches, whereas others do not. Also depending on the propensity for aggregation. It would be useful to elaborate this point. As disorder in itself is not a certain measure of disordered interactions, which can also be formed using structured domains, I suggest using some predictions of these interactions on selected positive and negative examples, and show the direct relationship between high AICAP scores and disordered interactions. Recently, the condensates could be predicted based on this feature, as described by Hahn (cell, 2018), Boija (cell, 2018)

**Response: We thank the reviewer for the suggestions. Indeed, there are various interactions within condensates, while hydrophobic interaction, which 1,6-HD is believed to disrupt, account for a subset of them. To better understand the relationship between AICAP and protein’s propensity to be involved in biomolecular condensates, we used common phase separation prediction tools to evaluate phase separation propensity of proteins with different AICAP values. As shown in Figure 2E of the revised manuscript, AICAP negatively correlates with FuzDrop score (PMID: 33318217). Similar but not as strong negative correlation were observed for PScore and PLAAC. AICAP of experiment using 2,5-HD as treatment didn’t reproduce similar results.**

**In terms of physicochemical properties of protein, comparison between AICAP and protein’s material state was conducted. Proteins reported to be liquid-like or hydrogel-like possess lower AICAP than average, while solid-like or amyloid fiber-forming proteins possess high AICAP on the contrary (Figure 3C).**

**Furthermore, after analysis the correlation between and AICAP and amino acid composition in specific protein region, we found that AICAP was negatively correlated with hydrophobic residue content (such as I and L) in IDR and PLD and positively correlated with positive charged residues (such as K and R) (Figure 2D). This relationship was not obvious or even opposite when we looked at the residue composition in whole protein sequence or ordered region.**

**Please see the descriptions on page 6-8 for the above updated analysis.**

Comment: The Discussion does not fit well with the rest of the paper. This is a method paper, which should serve as a general approach to study condensates. I expect the discussion about more the technical aspects, how this compares to other approaches,  whether biases for aggregation prone proteins etc, and much less about functional conclusions etc.

**Response: We thank the reviewer for the suggestion. In the revised discussion, we deleted most of the conclusions on protein function and written three new parts: 1). We systematically analyzed physicochemical properties of proteins with different AICAP values and compared AICAP with phase separation prediction tools, we found that AICAP is highly correlated with LLPS; 2). We discussed the difference between AICAP and FRAP analysis on specific proteins and discussed that AICAP may have advantages in testing protein dynamics under endogenous states and cellular concentrations; 3). We compared two recent reports on the role of LLPS in three-dimensional genomes. They reported that the active chromatin region was more affected to disturbance, which is consistent with our findings. See pages 12-13 of the revised manuscript for details.**

Comment: As condensates are formed by drivers and clients and their assembly/disassembly is regulated by a series of posttranslational modifications, I do not find these conclusions sufficiently founded. The community would benefit from discussing perspectives on systematic studies rather than making systems biology conclusions on novel, developing methodologies.

**Response: We thank the reviewer for mentioning the drivers and clients. However, there is still a lack of clear experimental evidence to distinguish drivers and clients. We extract list of self-assembling(drivers) and partner-dependent(clients) LLPS proteins based on experimental evidences from PhaSepDB database and examine their AICAP (Figure 3D). Both self-assembling and partner-dependent proteins display lower AICAP than average, while self-assembling proteins possess even lower AICAP. It suggests that self-assembling proteins are more dependent on hydrophobic interactions. See related descriptions on page 8 of the revised manuscript.**

**We agree that posttranslational modification (PTM) is important in condensate regulation. As regulation via PTM is one of the most sophisticated regulatory mechanism in cells, current AICAP value alone wouldn’t be able to draw a meaningful insight. We hope that when combined with phosphoproteomics, Hi-MS would provide valuable hints to reveal the roles of PTM in condensates in future study.**

Minor point

Comment: I suggest the authors to be a bit less technical and give a chance for a wider community to understand the approach and perspectives.

**Response: We agree with the opinion and re-organized the manuscript to be less technical and clearer.**

Comment: I also suggest to be careful with the examples, and compare to all possible data in literature, as some are controversial.

**Response: We agree that we should be careful with the examples. In this revision, we added the FRAP experiments of 7 proteins, which included FUSN, RNAPIICTD, H2B, DDX5, WDR43, PSPC1 and RBM14. FRAP results demonstrated that proteins with high mobility displayed low AICAP values (Figure 4). Furthermore, we compare the changes after 1,6-HD treatment to all possible data including widely used phase separation predictors, LLPS related features and ChIP-seq experiments. We discussed the difference between AICAP and FRAP analysis on specific proteins and discussed that AICAP may have advantages in testing protein dynamics under endogenous states and cellular concentrations. See sections from page 8-9.**

Comment: I think the work can be really valuable, if stays more method focused and discuss all the details related to methodology.

**Response: We thank the reviewer for the positive appreciation of our work and we agree that we should focus on method. Hope our revision make it much clearer.**

**Second round of review**

**Reviewer 1**

The authors have successfully addressed all my comments and I support publication of the manuscript in its current form.

**Reviewer 2**

The authors have done a nice job in addressing the comments and rewrite the ms to be more suitable for a broader audience interested in phase separation.
